# Supplementary material for: Control of STING Agonistic/Antagonistic Activity Using Amine-Skeleton-Based c-di-GMP Analogues
Source: Int J Mol Sci. 2022 Jun 20;23(12):6847. doi: 10.3390/ijms23126847 (PMC9224868; doi:10.3390/ijms23126847)
Supplement: Supplementary file 1 [file ijms-23-06847-s001.zip › ijms-1777081-supplementary.pdf]

Supporting Information

Control of STING agonistic/antagonistic activity using amine skeleton-based c-di-GMP analogues

Yuta Yanase,[1,3] Genichiro Tsuji,[1] Miki Nakamura,[1,2] Norihito Shibata,[1] and Yosuke Demizu*,[1,2,3]*

[1] *National Institute of Health Sciences, 3–25–26, Tonomachi, Kawasaki, Kanagawa, 210–9501, Japan*

[2] *Graduate School of Medicine Dentistry and Pharmaceutical Sciences, Division of Pharmaceutical Science of Okayama University, 1–1–1, Tsushimanaka, Kita, Okayama, 700–8530, Japan*

[3] *Graduate School of Medical Life Science, Yokohama City University, Yokohama, Kanagawa, 230–0045, Japan*

* *Correspondence: gtsuji@nihs.go.jp (G.T.); demizu@nihs.go.jp (Y.D.); Tel.: +81–44–270–6578*

Table of Contents

Supplementary Results

Figure S1 .... S4

Figure S2 .... S4

Figure S3 .... S3

Table S1 .... S3

Table S2 .... S4

Figure S4 .... S4

Figure S5 .... S5

Experimental procedures

Supplementary Results

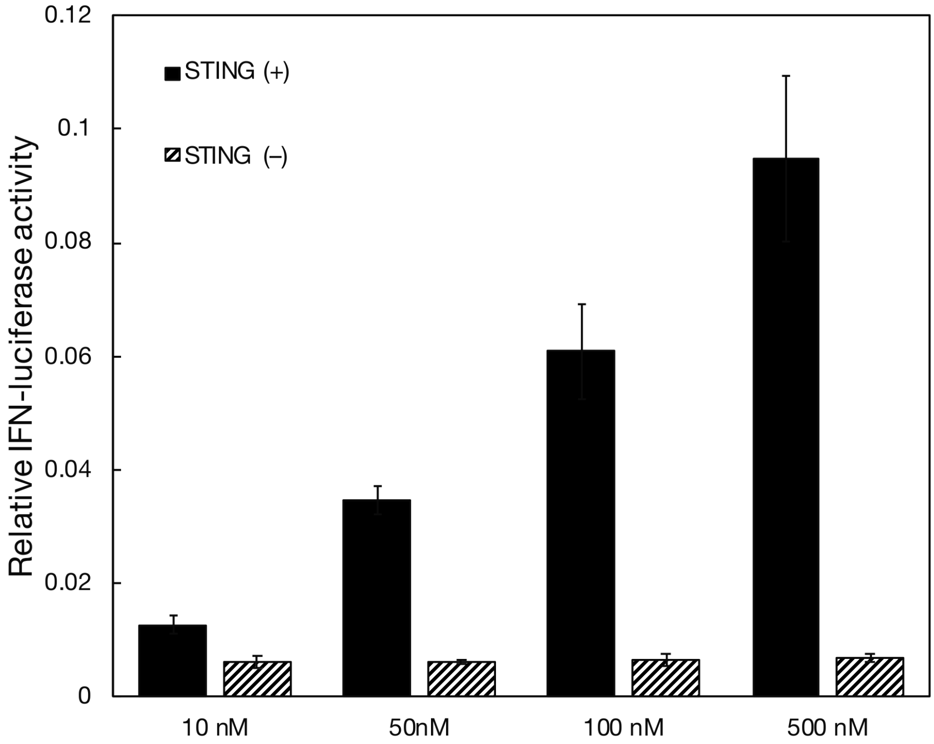

Figure S1. The evaluation of the IFN-β induction activity of cGAMP with/without STING transfection. HEK293T cells were transfected with vectors encoding Flag-STING or Flag-Empty as indicated, together with an IFN-luciferase reporter, and luciferase activity was measured 6 h after stimulation. Data are presented as mean ± SEM (*n* = 3).

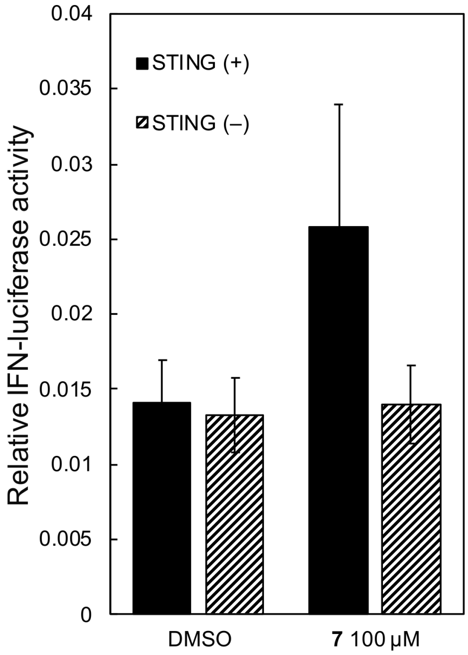

Figure S2. The evaluation of the IFN-β induction activity of **7** with/without STING transfection. HEK293T cells were transfected with vectors encoding Frag-STING or Flag-Empty as indicated, together with an IFN-luciferase reporter, and luciferase activity was measured 6 h after stimulation. Data are presented as mean ± SEM (*n* = 3).

Table S1. The evaluation of the IFN-β induction activity of cGAMP and **amine skeleton compounds** with digitonin permeabilization. HEK293T cells were transfected with vectors encoding STING as indicated, together with an IFN-luciferase reporter, and luciferase activity was measured at 6 h after stimulation.

Cpd. (100 μM)	Agonistic Activity	Antagonistic Activity
DMSO (1%)	0.040	N.A.
cGAMP (500 nM)	1.217	N.A.
**7**	0.075	0.97
**9**	0.045	1.41
**11**	0.039	1.53
**12**	0.045	0.78
**13**	0.048	1.42
**14**	0.043	1.24
**16**	0.040	1.40
**17**	0.042	1.29
**21**	0.045	1.74
**26**	0.047	1.12
**28**	0.038	1.52

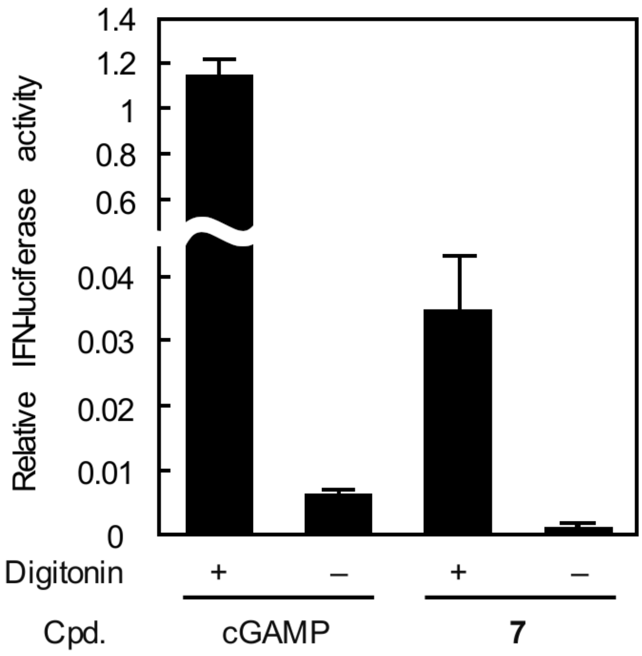

Figure S3. The evaluation of the IFN-β induction activity of cGAMP and **7** with/without digitonin permeabilization. HEK293T cells were transfected with vectors encoding STING as indicated, together with an IFN-luciferase reporter, and luciferase activity was measured 6 h after stimulation. IFN-luciferase activities were standardized by subtract the DMSO-treated control as background. Data are presented as mean ± SEM (n = 3).

Table S2. The evaluation of the IFN-β induction activity of cGAMP and **7** with/without digitonin permeabilization. HEK293T cells were transfected with vectors encoding STING as indicated, together with an IFN-luciferase reporter, and luciferase activity was measured 6 h after stimulation. IFN-luciferase activities were standardized by subtract the DMSO-treated control as background.

	cGAMP	Compound **7**
Digitonin –/+[a]	0.0056	0.037

[a] Relative value of luciferase activity of digitonin (-) to luciferase activity of digitonin (+)

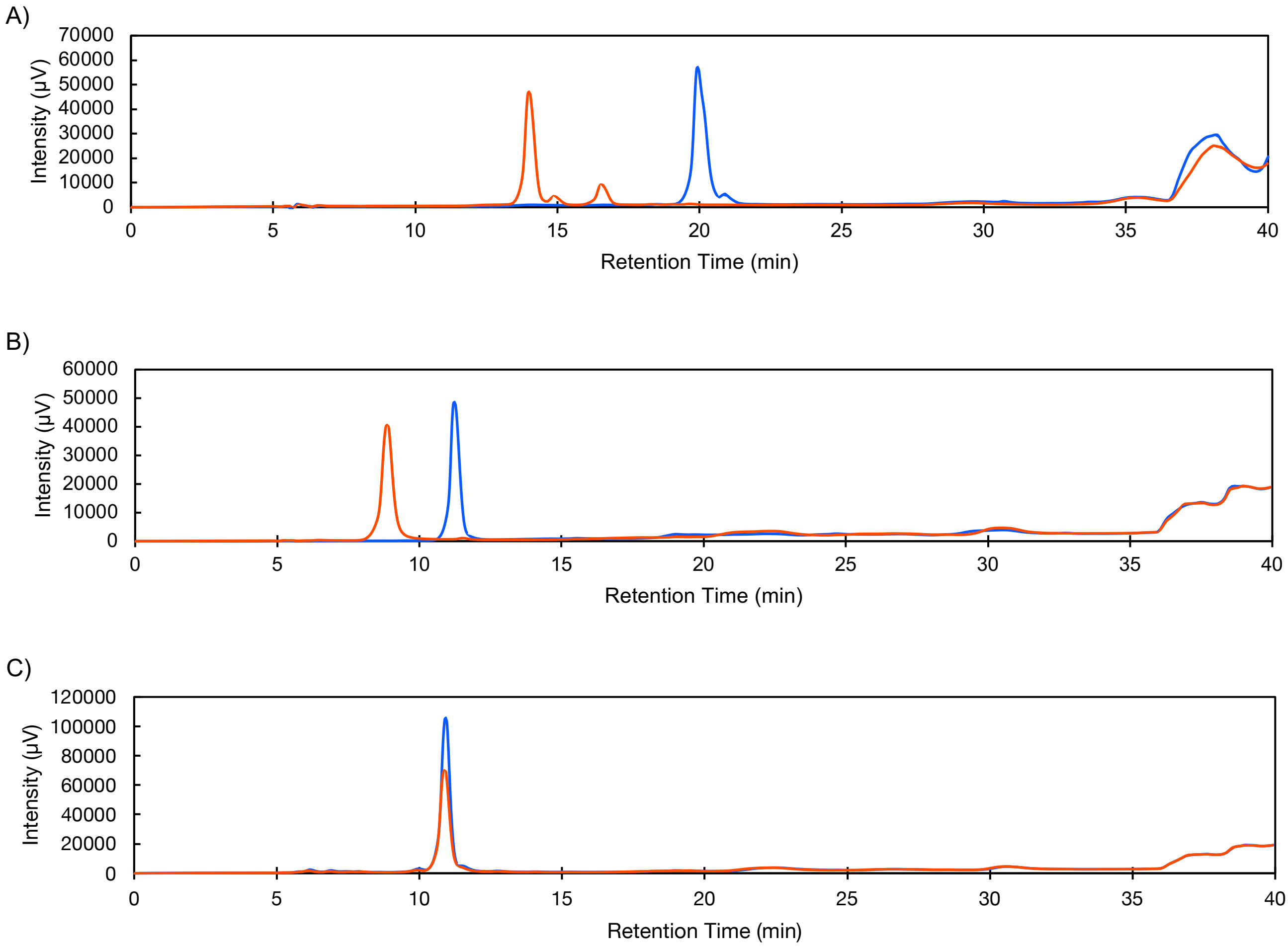

Figure S4. HPLC profile of the enzymatic digestion products of A) c-di-GMP, B) cGAMP, C) compound **7**. Red: NP1 (+), Blue: NP1(–).

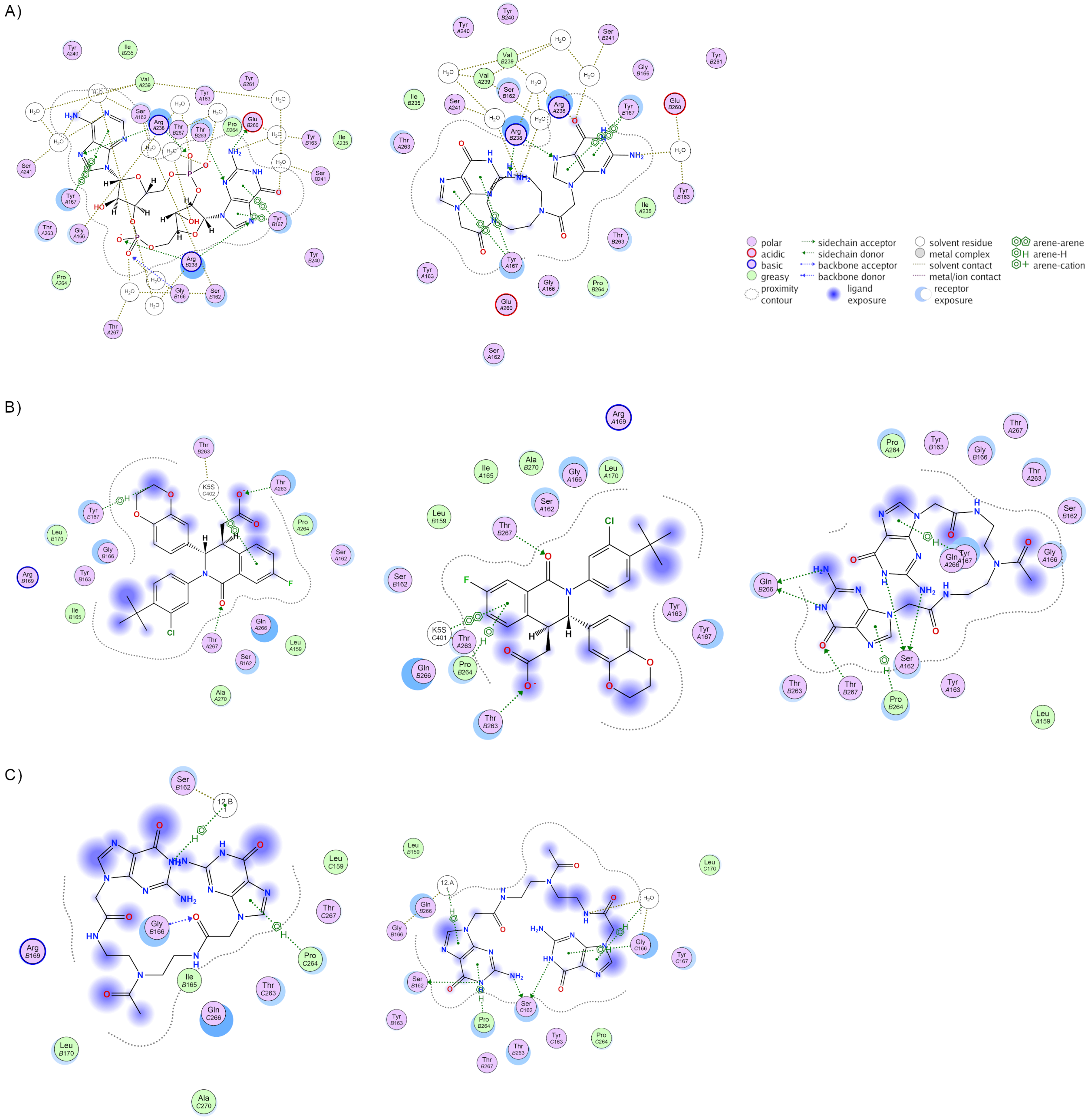

Figure S5. Docking study of **7** and **12** within the ligand binding site of STING (PDB: 4LOH for **7**, 6MXE for **12**) using MOE. A) 2D-maps of the essential amino acid residues interacting with A) cGAMP and **7,** B) two molecules of cpd. 18 and single molecule of **12**, C) two molecules of **12**. K5S B) shows the other molecule of cpd. 18. 12.A and 12.B in C) shows the other molecule of **12**.

Experimental procedures

Chemistry[S1]

General information

All chemicals were obtained from Sigma-Aldrich, FUJIFILM Wako Pure Chemical, Tokyo Chemical Industry Co., Ltd., and were used as received without further purification. TLC analysis, which was used to monitor the progress of reactions, was conducted using Merck silica gel 60 F254 pre-coated plates, visualizing with a 254/365 nm UV lamp and staining with iodine or ninhydrin. Column chromatography was performed using silica gel (spherical, neutral) purchased from Kanto Chemical or NH silica gel (CHROMATOREX NH-DM1020) purchased from Fuji Silysia, or medium pressure chromatography (Smart Flash; YAMAZEN) equipped with a Hi-Flash column and an Inject column (YAMAZEN). HPLC was performed using LC-2000Plus (JASCO). ^{1}H and ^{13}C NMR spectra were recorded on an ECZ 600R spectrometer (JEOL), and measurements were carried out using deuterated solvents. Chemical shift values (ppm) were calibrated using tetramethylsilane (TMS, 0.00 ppm) or residual non-deuterated solvent peaks as an internal reference ($CDCl_3$: 7.26 for ^{1}H NMR, 77.0 for ^{13}C NMR; CD_3OD: 3.30 for ^{1}H NMR, 49.0 for ^{13}C NMR; DMSO-d_6: 2.50 for ^{1}H NMR, 39.5 for ^{13}C NMR). Signal splitting patterns are described as singlet (s), doublet (d), triplet (t), quartet (q), quintet (quint.), septet (sept.), double of doublets (dd), doublet of triplet (dt), multiplet (m), broad (br.). High-resolution mass spectrum (HRMS) were measured using a Shimadzu IT-TOF MS equipped with an electrospray ionization source in positive mode or negative mode.

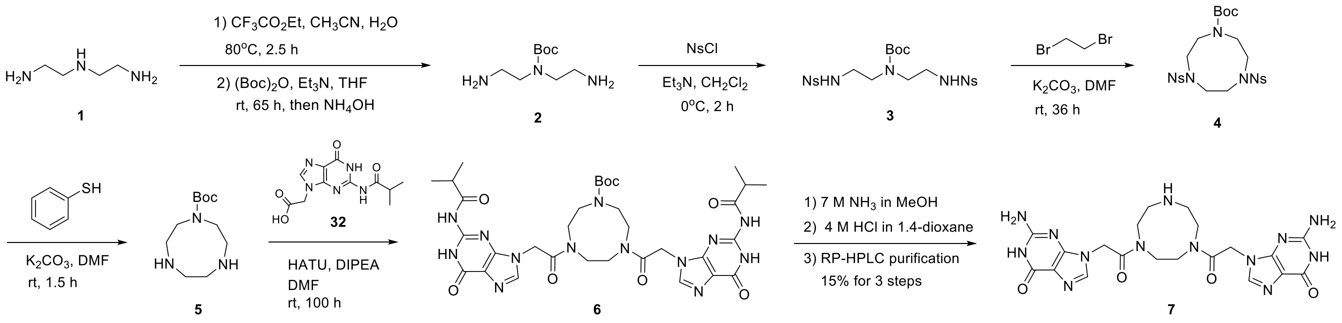

Scheme S1. Synthesis of compound **7**

tert-Butyl bis(2-(2,2,2-trifluoroacetamido)ethyl)carbamate (2)[S2]

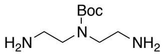

Diethylenetriamine (1.09 mL, 10 mmol) was dissolved in ACN (20 mL) containing water (200 µL). Ethyl trifluoroacetate (4.04 mL, 3.5 eq) was added to the solution, then the solution was refluxed for 14 h at 80ºC. The solvents were removed under reduced pressure, the residue was added 1:1 ACN – Et_2O (20 mL). The solvents were removed under reduced pressure, dried under vacuum. The resulting residue was dissolved in THF (7 mL). Et_3N (2.09 mL, 1.5 eq) and $(Boc)_2O$ (3.31 g, 1.5 eq) in THF (3 mL) was added dropwise into the solution under stirring at 0ºC. The mixture was stirred over weekend at rt. Almost solvents were removed under reduced pressure. The resulting residue was dissolved in 28% NH_4OH (10 mL) and MeOH (5 mL), stirred over weekend at 50ºC. The reaction mixture was concentrated under reduced pressure, purified by flash silica gel column chromatography (φ = 42 mm, h = 145 mm, CH_2Cl_2/MeOH/28% NH_4OH = 20:5:1) to afford a pale yellowish oil including **2** (4.63 g). This oil was used next reaction without further purification.

^{1}H NMR (600 MHz, DMSO-d_6) δ 7.77 (br.s, 4H), 2.92 (t, J = 7.5 Hz, 4H), 1.41 (s, 9H). ESI-HRMS calcd for $C_9H_{22}N_3O_2$ $[M+H]^+$: 204.1707, found : 204.1716.

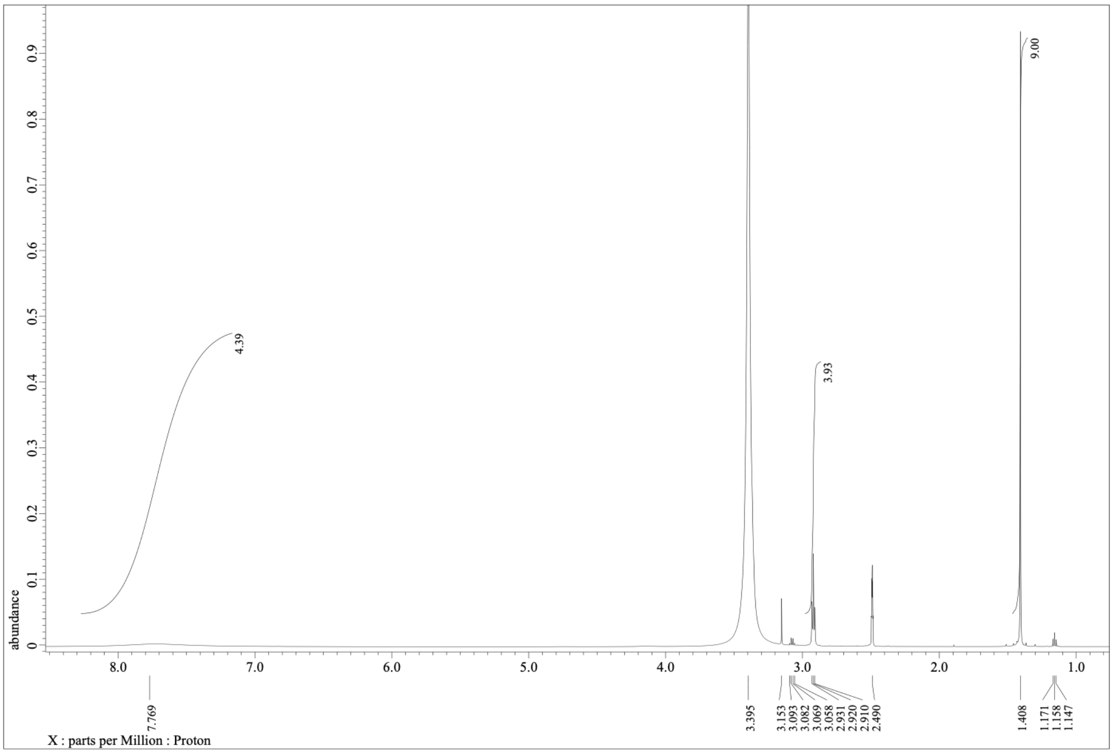

^{1}H NMR spectrum of **2**

tert-Butyl bis(2-(2-nitrophenylsulfonamido)ethyl)carbamate (3)

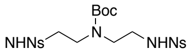

The oil including **2** (203 mg) was dissolved in CH_2Cl_2 (5 mL), added Et_3N (613 µL, 4.4 eq) at rt. 2-Nitrobenzensulfonyl chloride (487.5 mg, 2.2 eq) was added into the mixture under stirring at 0ºC, the mixture was continue stirring for 2 h at 0ºC. The reaction mixture was diluted with CH_2Cl_2, washed with $KHSO_4$ aq. (2 M), brine. The organic layer was dried over Na_2SO_4, filtered, concentrated under reduced pressure. The residue was purified by flash silica gel column chromatography (Yamazen inject column size: M, Yamazen Hi-flash column size: M, CH_2Cl_2/MeOH = 100:0 to 97:3) to afford **3** (169 mg, 0.3 mmol, 68% yield for 4 steps) as a white foam solid.

^{1}H NMR (600 MHz, DMSO-d_6) δ 8.13 (dt, *J* = 5.7 Hz, 24 Hz, 2H), 7.94-7.97 (m, 4H), 7.84-7.86 (m, 4H), 3.17 (d, *J* = 6.6 Hz, 4H), 2.98 (q, *J* = 6.6 Hz, 4H), 1.33 (s, 9H). ESI-HRMS calcd for $C_{21}H_{28}N_5O_{10}S_2$ [M-H]$^-$: 572.1126, found: 572.1082.

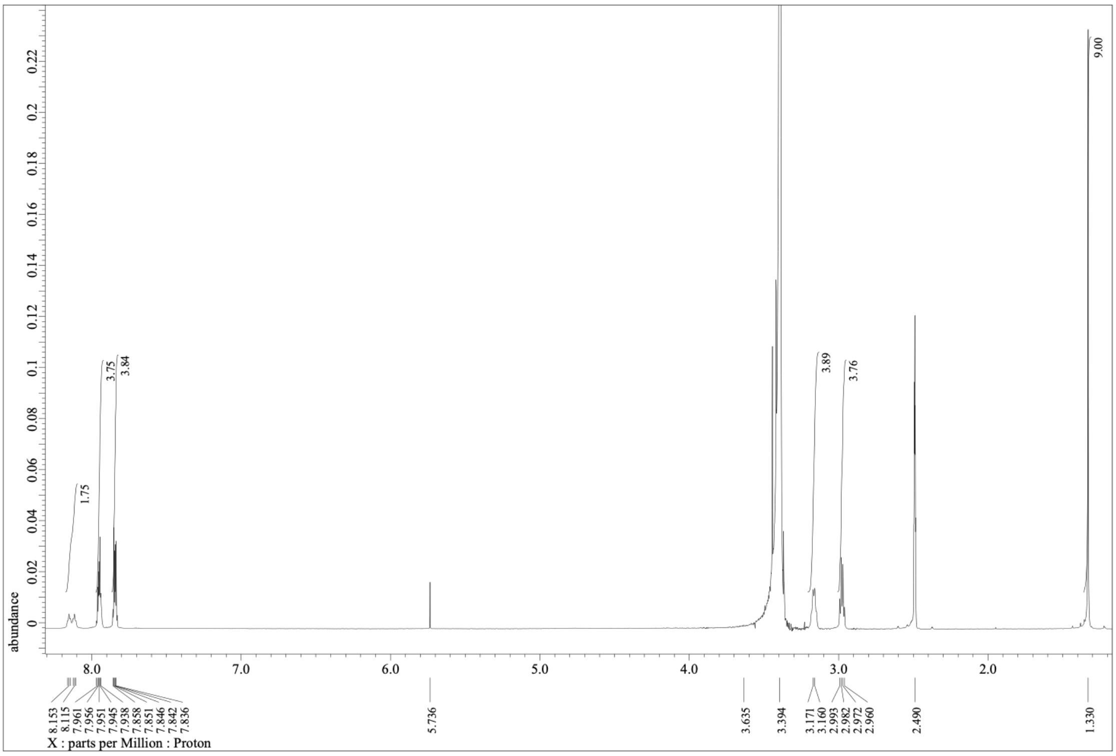

^{1}H NMR spectrum of **3**

tert-Butyl 4,7-bis((2-nitrophenyl)sulfonyl)-1,4,7-triazonane-1-carboxylate (4)

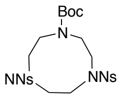

Compound **3** (229 mg, 0.4 mmol) was dissolved in DMF (1 mL), added K_2CO_3 (110 mg, 2 eq) at rt then stirred at 0ºC. 1,2-dibromoethane (75 mg, 1 eq) in DMF (1 mL) was added dropwise into the mixture at 0ºC. The mixture was stirred for overnight at rt. K_2CO_3 (55 mg, 1 eq) and 1,2-dibromoethane (75 mg, 1 eq) was added into the mixture, stirred for over weekend. The reaction mixture was diluted with EtOAc, washed with HCl (2 M) twice, sat. aq. $NaHCO_3$, brine. The organic layer was dried over Na_2SO_4, filtered, concentrated under reduced pressure. The residue was purified by NH silica gel column chromatography (φ = 15 mm, h = 105 mm, CH_2Cl_2/MeOH = 100:0 to 98:2) to afford **4** (150 mg, 0.25 mmol, 63% yield) as a pale yellow foam.

^{1}H NMR (600 MHz, $CDCl_3$) δ 7.62-8.05 (m, 8H), 3.27-3.73 (br.m, 12H), 1.44-1.50 (m, 9H); ^{13}C NMR (151 MHz, $CDCl_3$) δ 155.5, 148.6. 148.6, 134.0, 133.9, 131.8, 131.7, 130.6, 130.5, 124.2, 80.8, 53.4, 52.7, 51.7, 51.3, 50.9, 45. 7, 28.3. ESI-HRMS calcd for $C_{11}H_{22}NO_2$ $[M-H]^-$: 598.1283, found: 598.1246.

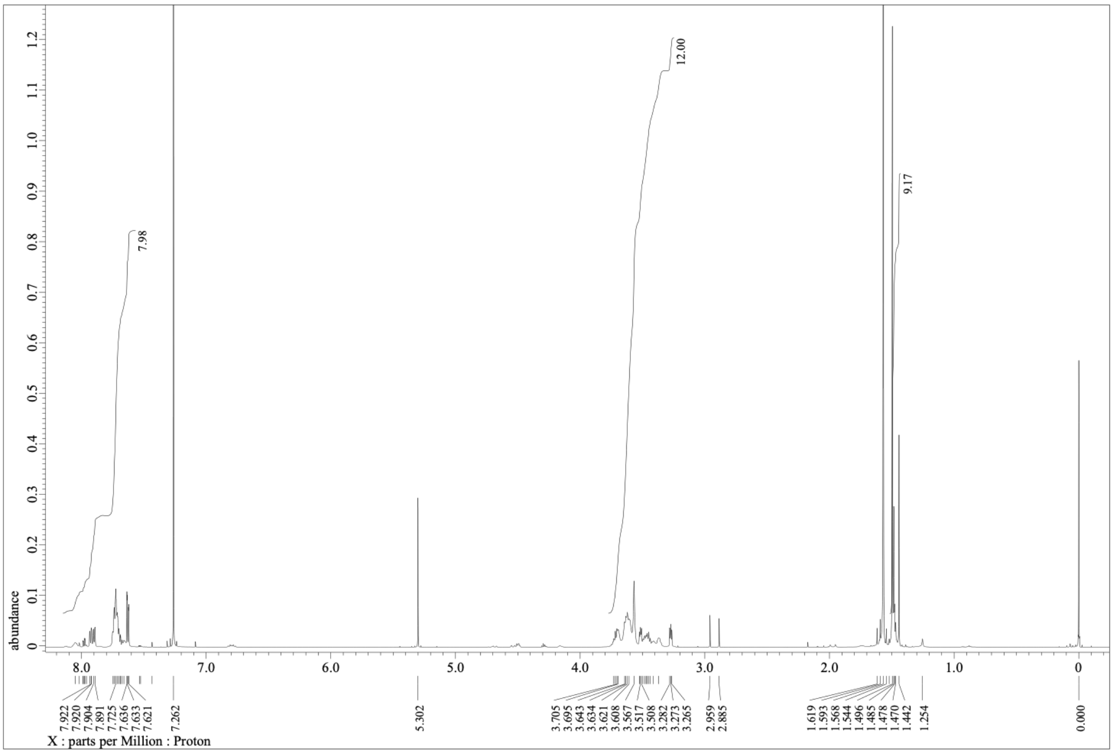

^{1}H NMR spectrum of **4**

***tert*-Butyl 1,4,7-triazonane-1-carboxylate (5)**

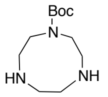

Compound **4** (150 mg, 0.25 mmol) was dissolved in DMF (1.2 mL), added K_2CO_3 (111 mg, 3.2 eq) at rt then stirred at 0ºC. Thiophenol (82 μL, 3.2 eq) was added into the mixture at 0ºC, stirred for 1.5 h at rt. The reaction mixture was diluted with CH_2Cl_2, filtered, concentrated under reduced pressure, dried under vacuum. The residue was purified by NH silica gel column chromatography (φ = 25 mm, h = 70 mm, CH_2Cl_2/MeOH = 100:0 to 92:8) to afford **5** (47.4 mg, 0.21 mmol, 82% yield) as a yellow foam.

^{1}H NMR (600 MHz, $CDCl_3$) δ 4.29-2.70 (m, 12H), 1.49-1.43 (m, 9H). ESI-HRMS calcd for $C_{11}H_{24}N_3O_2$ $[M+H]^+$: 230.1863, found: 230.1863.

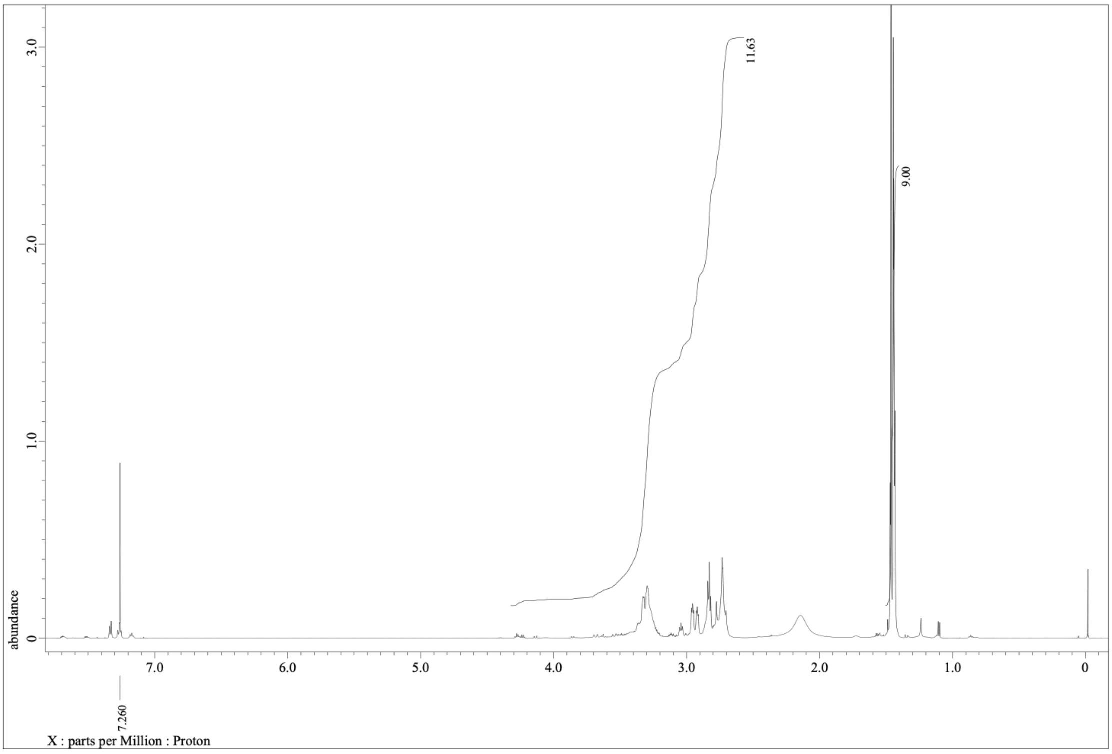

^{1}H NMR spectrum of **5**

2-Amino-9-(2-(4-(2-(2-amino-6-oxo-1*H*-purin-9(6*H*)-yl)acetyl)-1,4,7-triazonan-1-yl)ethyl)-1*H*-purin-6(9*H*)-one (7)

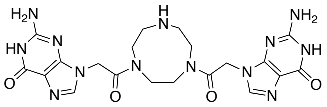

Compound **5** (47.4 mg, 0.21 mmol) was dissolved in DMF (1 mL) and stirred at 0ºC. Compound **32** (126 mg, 2.2 eq), HATU (236 mg, 3 eq) and DIPEA (216 µL, 6 eq) was added into the above mixture, stirred for over weekend at rt. The reaction mixture was concentrated under reduced pressure.

The resulting residue was added NH_3 in MeOH (7 M, 1 mL), stirred for overnight at rt. The mixture was added additional NH_3 in MeOH (7 M, 2 mL), stirred for over weekend at rt. The reaction mixture was concentrated under reduced pressure, dried under vacuum.

The resulting residue was added HCl in 1,4-dioxane (4 M, 3 mL), stirred for 2 h at rt. The reaction mixture was concentrated under reduced pressure, the residue was dried by codistillation with MeOH twice. The residue was purified by HPLC (LC-2000Plus (JASCO); Discovery BIO Wide Pore C18 HPLC Column (Supelco), 21.2 × 250 mm; Flow rate: 10 mL/min; 40ºC; Mobile phase A: 0.1% TFA in H_2O, B: 0.1% TFA in ACN; Gradient: B 0% to 10 % (20 min); Detection: 260 nm) to afford **7** (15.8 mg, 0.031 mmol, 14.7% yield for 3 steps) as a white solid.

^{1}H NMR (600 MHz, DMSO-*d₆*) δ 10.18-10.05 (m, 2H), 7.30-7.22 (m, 2H), 6.01-5.56 (m, 4H), 4.12 (s, 1H), 4.02 (s, 1H), 3.94 (s, 1H), 3.83 (s, 1H), 2.74-2.56 (m, 2H), 2.46 (s, 2H), 2.20-2.13 (m, 4H), 2.02 (s, 1H); ^{13}C NMR (151 MHz, DMSO-d_6) δ 167.6, 167.4, 166.1, 158.9, 158.7, 158.4, 158.2, 155.2, 155.0, 154.9, 154.8, 154.6, 150.9, 150.8, 150.7, 150.7, 150.6, 138.7, 138.5, 138.4, 118.5, 116.6, 114.7, 112.7, 110.5, 46.4, 46.0, 45.3, 45.2, 43.6, 43.4. ESI-HRMS calcd for $C_{20}H_{26}N_{13}O_4$ [M+H]$^+$: 512.2225, found: 512.2253.

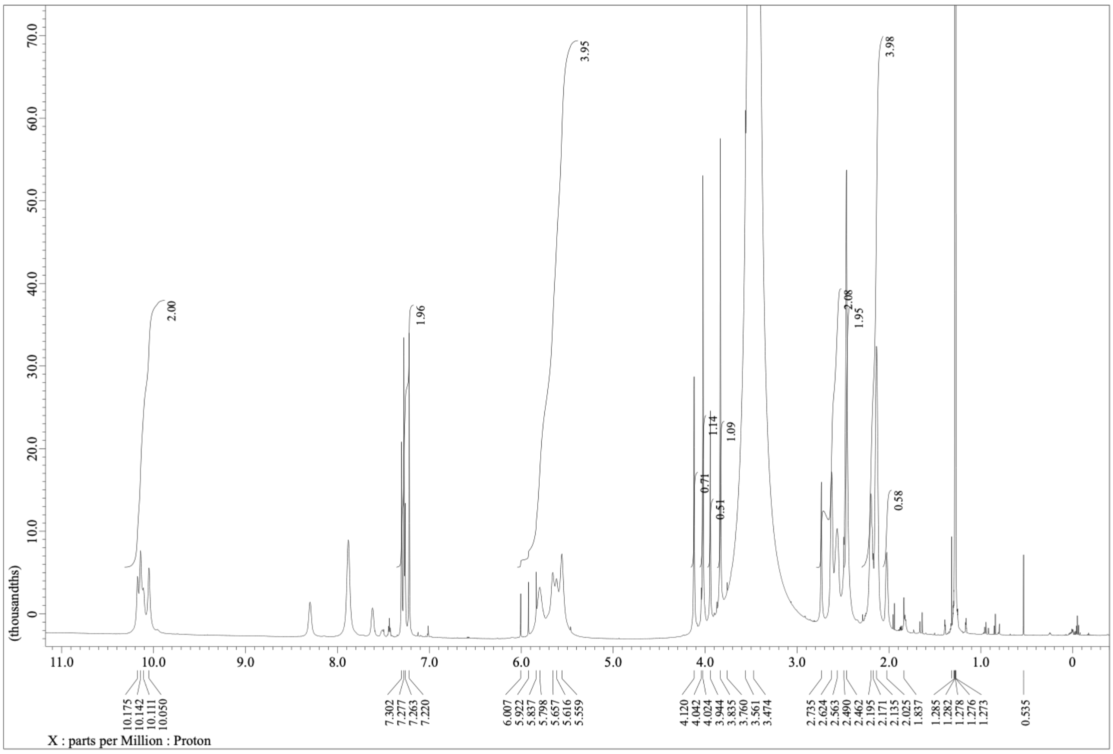

^{1}H NMR spectrum of **7**

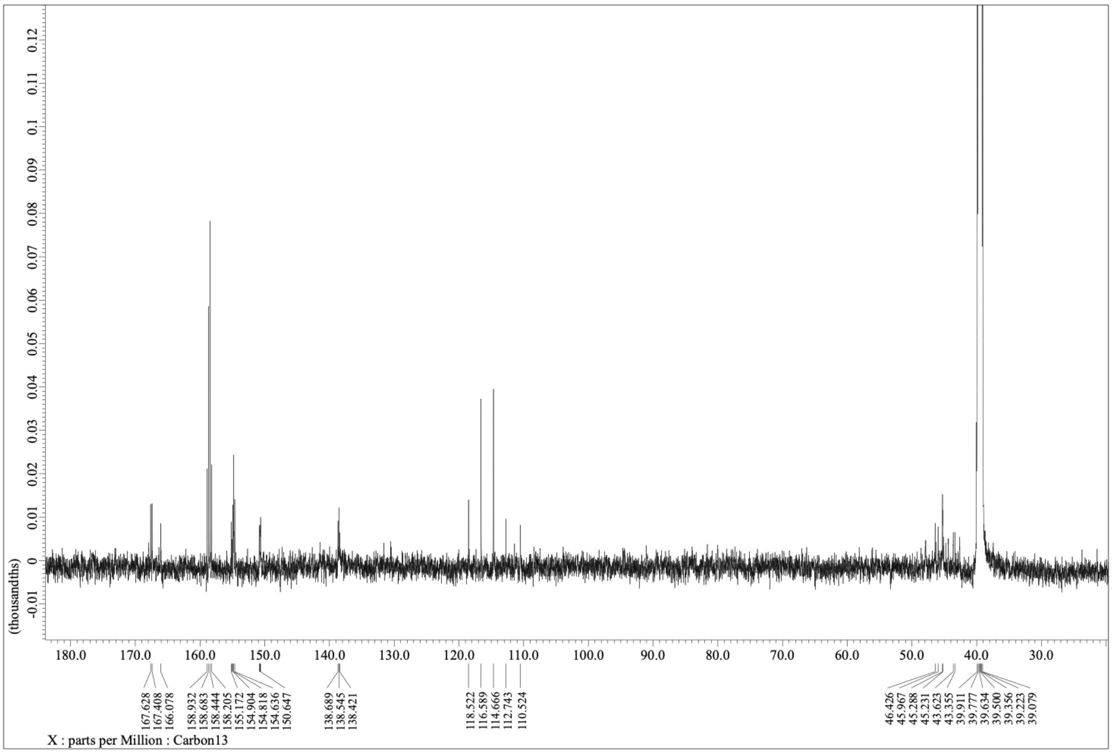

^{13}C NMR spectrum of **7**

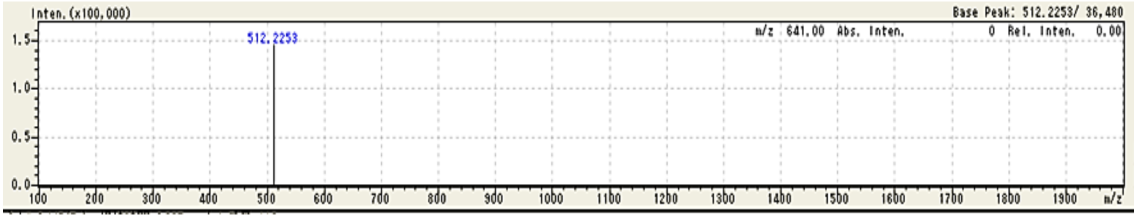

ESI-MS spectrum of **7**

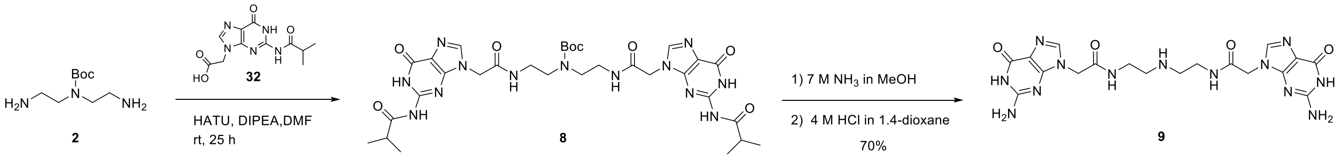

Scheme S2. Synthesis of compound **9**

tert-Butyl bis(2-(2-(2-isobutyramido-6-oxo-1_H_-purin-9(6_H_)-yl)acetamido) ethyl)carbamate (8)

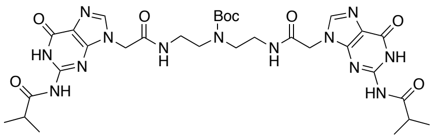

The oil including **2** (203 mg) was dissolved in DMF (5 mL) and stirred at 0ºC. Compound **32** (614 mg, 2.2 eq), HATU (1.14 g, 3 eq) and DIPEA (1 mL, 6 eq) was added into the mixture, stirred for 23 h at rt. The reaction mixture was diluted with EtOAc, washed with HCl (1 M), brine. The aqueous layer was extracted 3 times with CH_2Cl_2. The organic layers were dried over Na_2SO_4, filtered, concentrated under reduced pressure, dried under vacuum. The crude mixture was absorbed on silica gel (Yamazen inject column size: M, h = 2 cm).

Purified by flash silica gel column chromatography (Yamazen Hi-flash column size: M, CH_2Cl_2/MeOH = 99:1 to 50:50) to afford **8** (117 mg, 0.16 mmol, 37% yield for 4 steps) as a pale yellow foam solid.

^{1}H NMR (600 MHz, CD_3OD) δ 8.30 (br.s, 1H), 7.99 (s, 2H), 3.42-3.31 (br.m, 8H), 2.66 (br.s, 2H), 1.46 (s, 9H), 1.18 (d, J = 6.9 Hz, 12H); ^{13}C NMR (151 MHz, CD_3OD) δ 181.7, 168.9, 157.6, 151.2, 149.7, 142.3, 120.7, 81.7, 39.6, 36.9, 28.8, 19.3. ESI-HRMS calcd for $C_{31}H_{44}N_{13}O_8$ $[M+H]^+$: 726.3430, found: 726.3423.

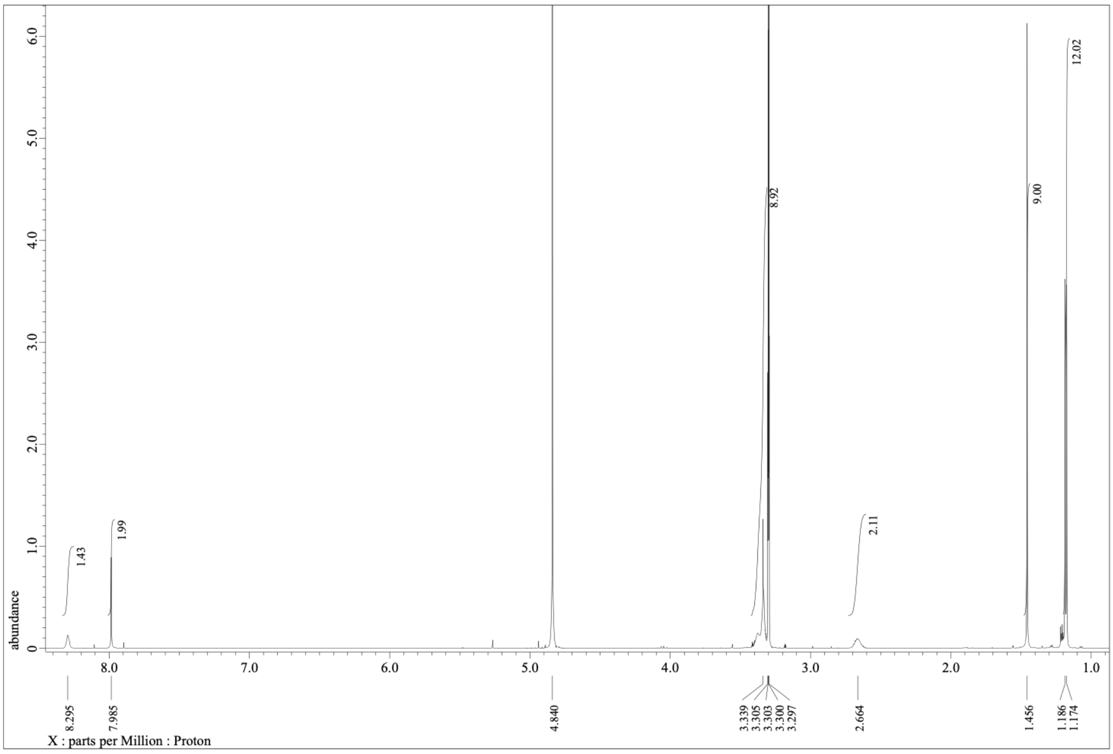

^{1}H NMR spectrum of **8**

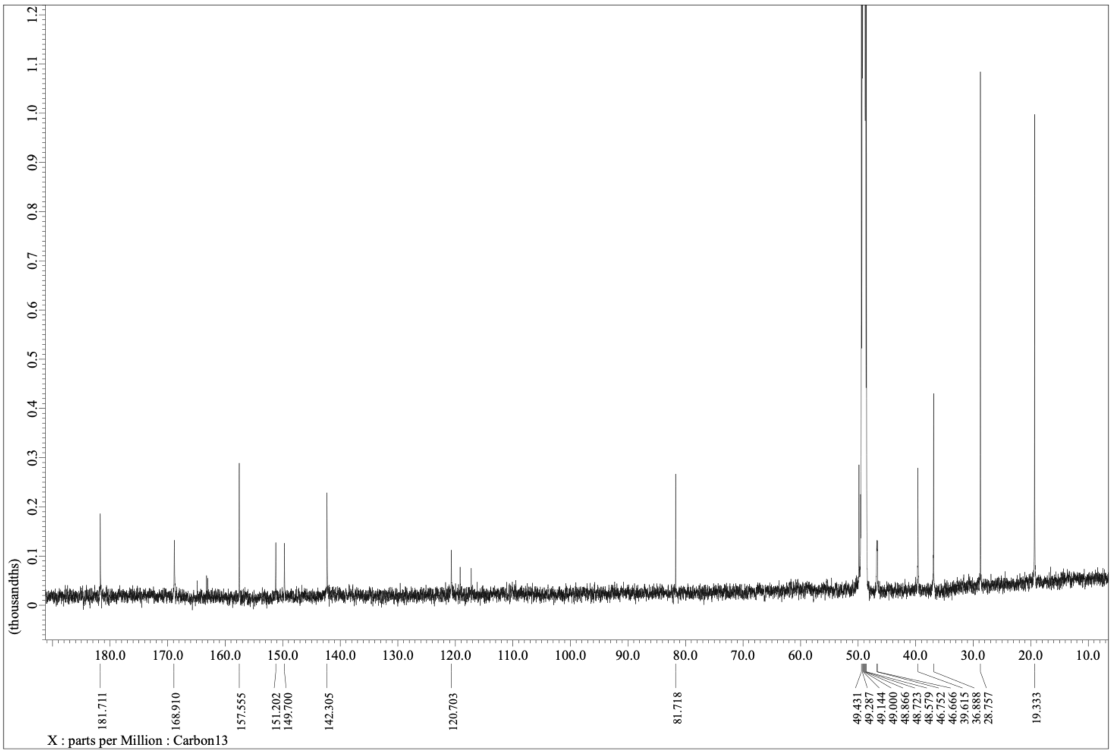

^{13}C NMR spectrum of **8**

_N_,_N'_-(Azanediylbis(ethane-2,1-diyl))bis(2-(2-amino-6-oxo-1_H_-purin-9(6_H_)-yl)acetamide) (9)

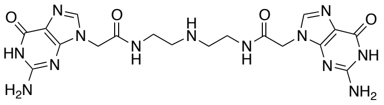

Compound **8** (10 mg, 0.014 mmol) was dissolved in NH_3 in MeOH (7 M, 1 mL), stirred over weekend at rt. The resulting precipitate was collected by centrifugation, washed with ACN, dried under vacuum.

The residue was added HCl in 1,4-dioxane (4 M, 1 mL), stirred for 2 h at rt. The reaction mixture was concentrated under reduced pressure and the residue was dried by codistillation with MeOH to afford **9** (5.8 mg, 9.8 μmol, 70% yield) as a pale yellow solid.

^{1}H NMR (600 MHz, DMSO-d_6) δ 11.49 (s, 2H), 9.31 (s, 2H), 8.84 (t, J = 5.5 Hz, 2H), 8.78 (s, 2H), 7.13 (s, 4H), 4.85 (s, 4H), 3.49-3.44 (m, 4H), 3.04 (t, J = 5.9 Hz, 4H); ^{13}C NMR (151 MHz, DMSO-d_6) δ 166.0, 155.1, 154.4, 150.4, 138.2, 131.6, 128.7, 109.8, 94.7, 72.2, 70.5, 70.5, 69.6, 66.4, 60.2, 48.6, 45.9, 45.8, 43.6, 35.3. ESI-HRMS calcd for $C_{18}H_{24}N_{13}O_4$ $[M+2H]^{2+}$: 243.6071, found: 243.6134.

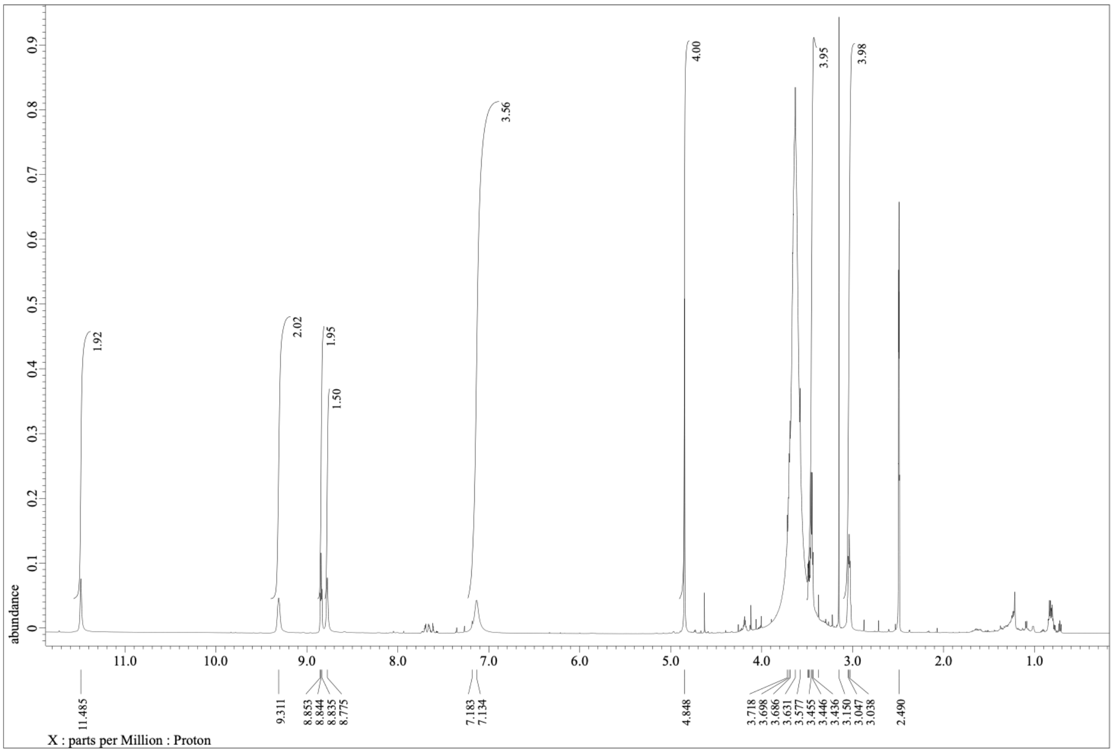

^{1}H NMR spectrum of **9**

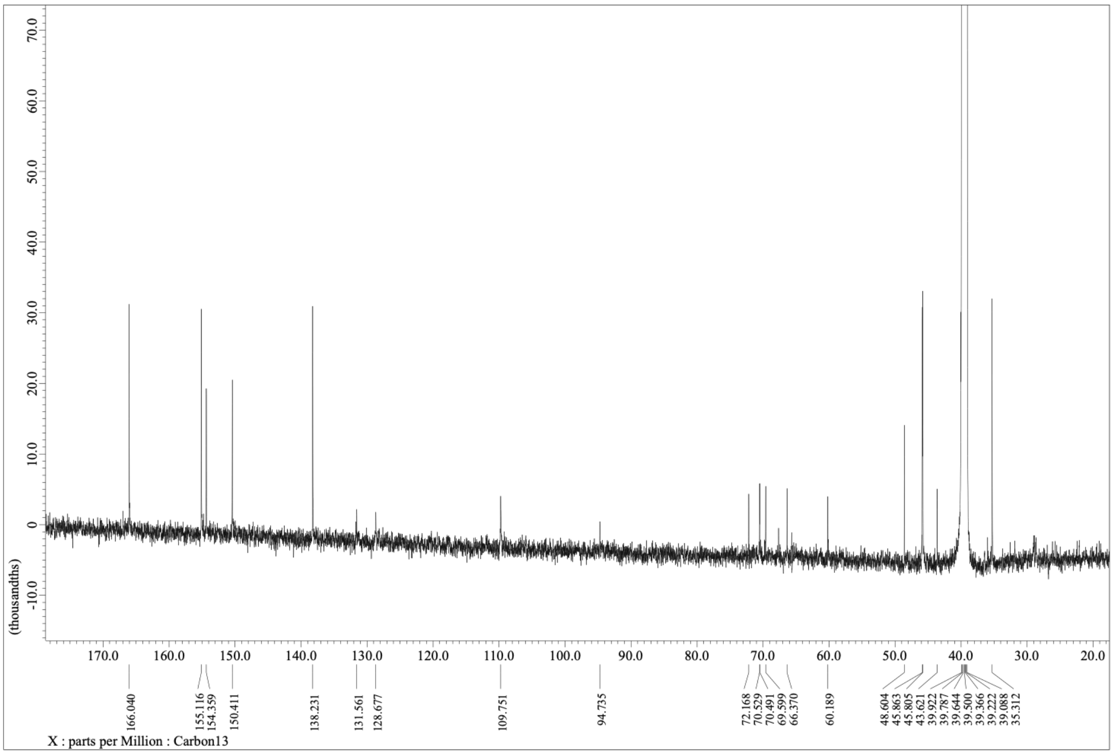

^{13}C NMR spectrum of **9**

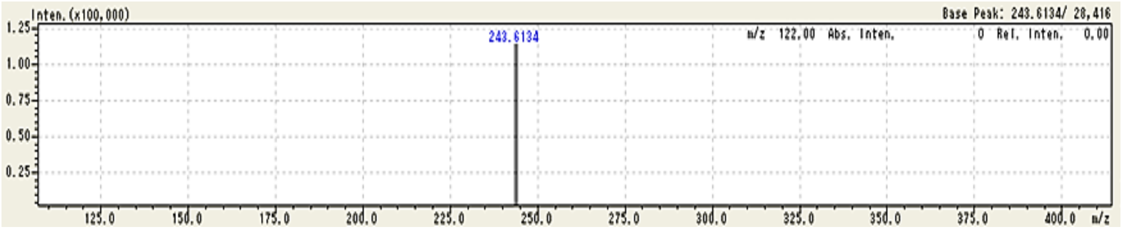

ESI-MS chromatogram of **9**

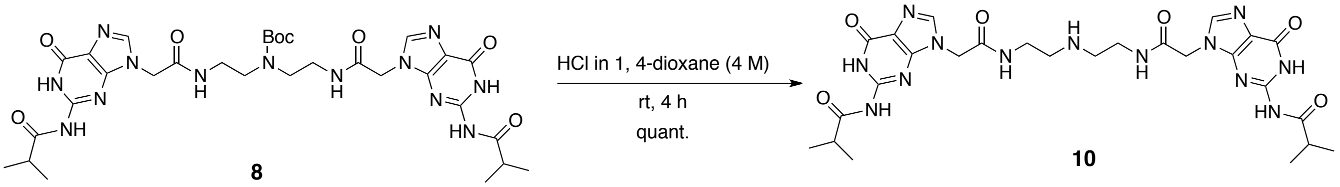

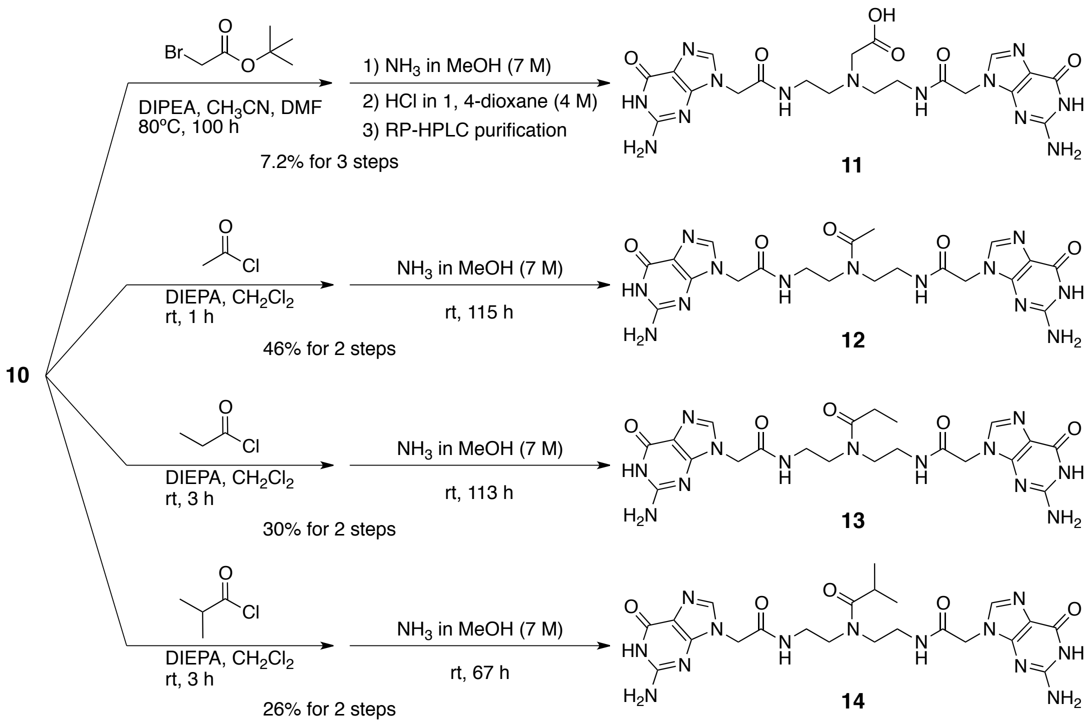

Scheme S3. Synthesis of compounds **11**, **12**, **13** and **14**

***N*,*N*'-(9,9'-(((Azanediylbis(ethane-2,1-diyl))bis(azanediyl))bis(2-oxoethane-2,1-diyl))bis(6-oxo-6,9-dihydro-1*H*-purine-9,2-diyl))bis(2-methylpropanamide) (10)**

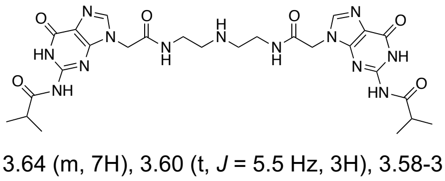

Compound **8** (63 mg, 0.087 mmol) was dissolved in HCl in 1,4-dioxane (4 M, 10 mL), stirred for 1.5 h at rt. The reaction mixture was concentrated under reduced pressure and the residue was dried by codistillation with MeOH to afford **10** (59.7 mg, 0.090 mmol, quant.) as a yellow solid.

^{1}H NMR (600 MHz, CD_3OD) δ 8.93 (s, 2H), 5.08 (s, 3H), 3.73 (t, *J* = 5.9 Hz, 3H), 3.67-3.64 (m, 7H), 3.60 (t, *J* = 5.5 Hz, 3H), 3.58-3.56 (m, 3H), 3.24 (t, *J* = 5.5 Hz, 3H), 2.71 (sept., *J* = 6.7 Hz, 2H), 1.22 (d, *J* = 6.9 Hz, 12H). ESI-HRMS calcd for $C_{26}H_{36}N_{13}O_6$ $[M+H]^+$: 626.2906, found: 626.2862.

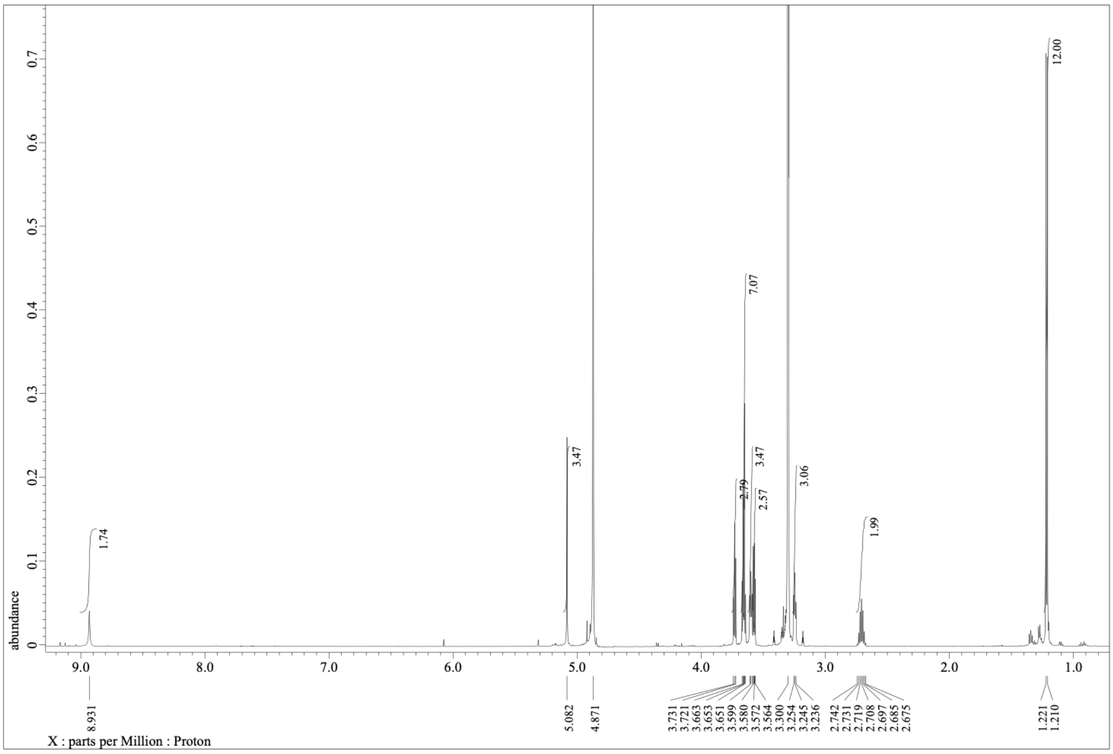

^{1}H NMR spectrum of **10**

2-(Bis(2-(2-(2-amino-6-oxo-1*H*-purin-9(6*H*)-yl)acetamido)ethyl)amino) acetic acid (11)

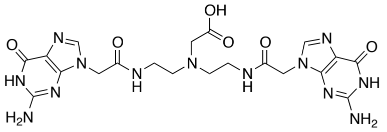

Compound **10** (91 mg, 0.14 mmol) was dissolved in ACN (1.4 mL) and DMF (1.4 mL), then stirred at rt. DIPEA (141 µL, 5.8 eq) and *tert*-butyl 2-bromoisobutyrate (50 µL, 2.4 eq) was added into the mixture, stirred for over weekend at 80ºC. The reaction mixture wan concentrated under reduced pressure, diluted with EtOAc, washed with HCl (1 M), sat. $NaHSO_4$ aq., brine. The combined aqueous layers were concentrated under reduced pressure, dried by codistillation with ACN. The residue was added NH_3 in MeOH (7 M, 10 mL), stirred for overnight at 40ºC. The reaction mixture was concentrated under reduced pressure. The residue was added HCl in 1,4-dioxane (4 M, 10 mL), stirred for 5 h at rt. The reaction mixture was concentrated under reduced pressure, the residue was dried by codistillation with ACN. The residue was purified by HPLC (LC-2000Plus (JASCO); Discovery BIO Wide Pore C18 HPLC Column (Supelco), 21.2 × 250 mm, 10 µm; Flow rate: 10 mL/min; rt; Mobile phase A: 0.1% TFA in H_2O, B: 0.1% TFA in ACN; Gradient: B 0% to 30 % (20 min); Detection: 260 nm) to afford **11** (8.5 mg, 0.016 mmol, 7.2% yield for 3 steps) as a pale yellow solid.

^{1}H NMR (600 MHz, DMSO-d_6) δ 10.87 (s, 2H), 8.42 (s, 2H), 7.95 (s, 2H), 6.62 (s, 4H), 4.68 (s, 4H), 3.98 (br. s, 2H), 3.39 (br. d, *J* = 5.5 Hz, 4H), 3.13 (s, 4H); ^{13}C NMR (151 MHz, DMSO-d_6) δ 167.0, 158.6, 158.4, 158.1, 157.9, 155.9, 154.1, 151.1, 138.2, 56.0, 53.8, 52.6, 45.7, 45.1, 18.7. ESI-HRMS calcd for $C_{20}H_{26}N_{13}O_6$ $[M+H]^+$: 544.2124, found: 544.2074.

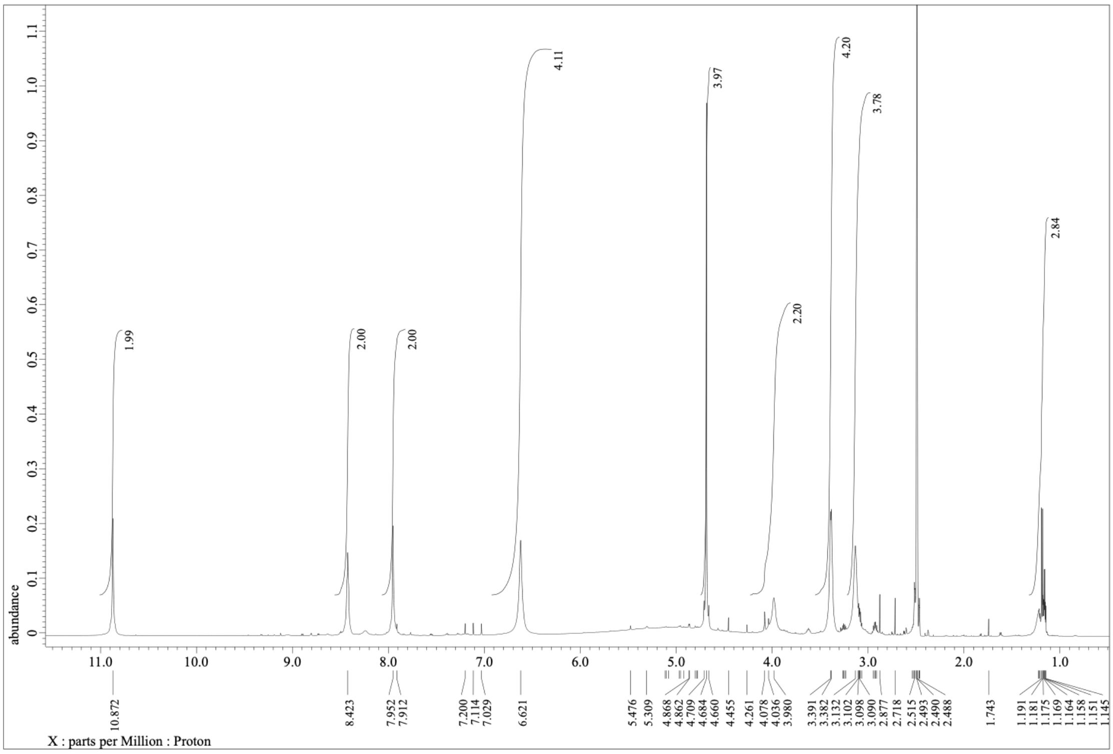

^{1}H NMR spectrum of **11**

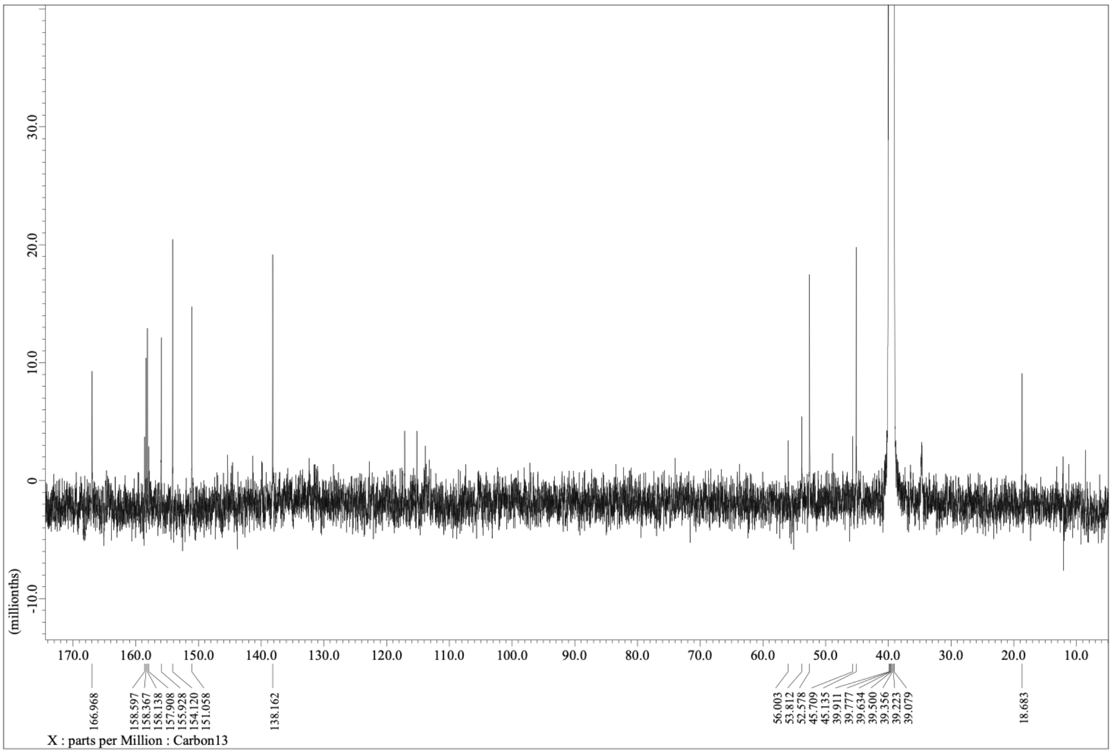

^{13}C NMR spectrum of **11**

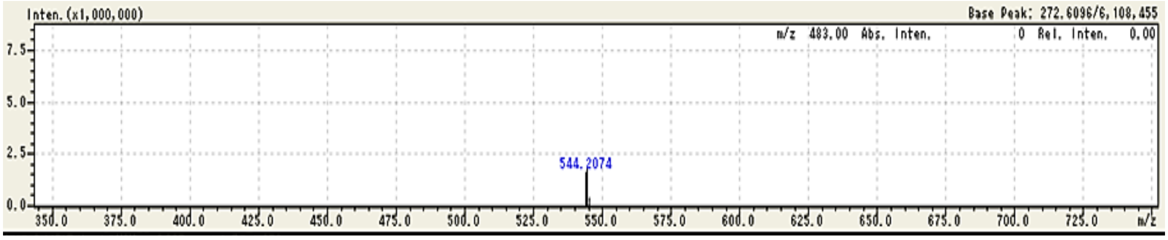

ESI-MS chromatogram of **11**

2-(2-Amino-6-oxo-1*H*-purin-9(6*H*)-yl)-*N*-(2-(*N*-(2-(2-(2-amino-6-oxo-1*H*-purin-9(6*H*)-yl)acetamido)ethyl)acetamido)ethyl)acetamide (12)

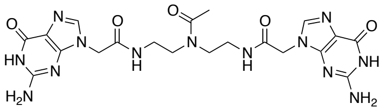

Compound **10** (72 mg, 0.1 mmol) was dissolved in CH_2Cl_2 (1 mL) and DIPEA (95 µL, 5 eq), then stirred at 0ºC. Acetyl chloride (16 µL, 2 eq) was added into the mixture, stirred for 1.5 h at rt. The reaction mixture was concentrated under reduced pressure, dried under vacuum. The residue was added NH_3 in MeOH (7 M, 10 mL), stirred over weekend at rt, added additional NH_3 in MeOH (7 M, 5 mL), stirred for 6 h at 40ºC. The reaction mixture was concentrated under reduced pressure. The residue was added NH_3 in MeOH (7 M, 15 mL), stirred for 23 h at 50ºC. The reaction mixture was concentrated under reduced pressure. The residue was added MeOH, filtered, washed with MeOH, dried under vacuum to afford **12** (24.1 mg, 0.046 mmol, 46% yield for 2 steps) as a white solid.

^{1}H NMR (600 MHz, DMSO-*d*$_6$) δ 8.23 (dt, *J* = 51.6, 5.5 Hz, 2H), 7.58 (d, *J* = 1.4 Hz, 2H), 6.42 (d, *J* = 6.2 Hz, 4H), 4.59 (d, *J* = 19.3 Hz, 4H), 3.30-3.16 (m, 4H), 1.97 (s, 3H); ^{13}C NMR (151 MHz, DMSO-*d*$_6$) δ 170.3, 167.0, 166.7, 156.9, 153.6, 151.5, 138.3, 138.2, 116.2, 116.2, 47.8, 44.9, 44.9, 44.7, 37.5, 36.8, 21.3. ESI-HRMS calcd for $C_{20}H_{26}N_{13}O_5$ [M+H]$^+$: 528.2174, found: 528.2299.

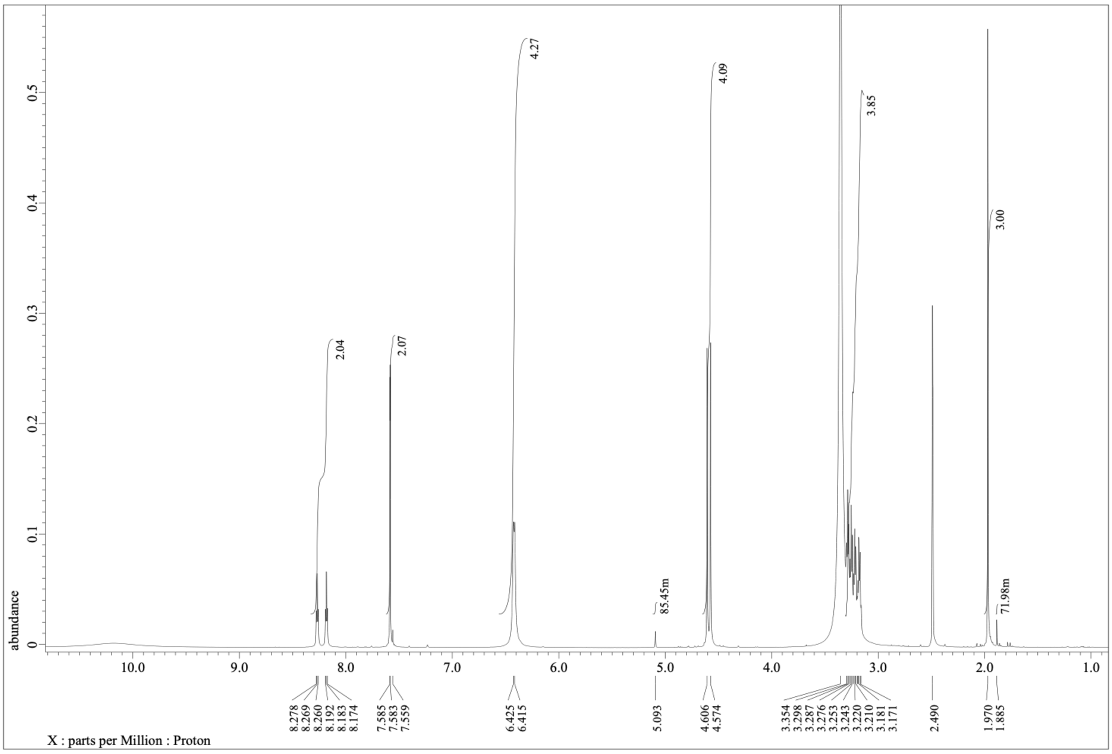

^{1}H NMR spectrum of **12**

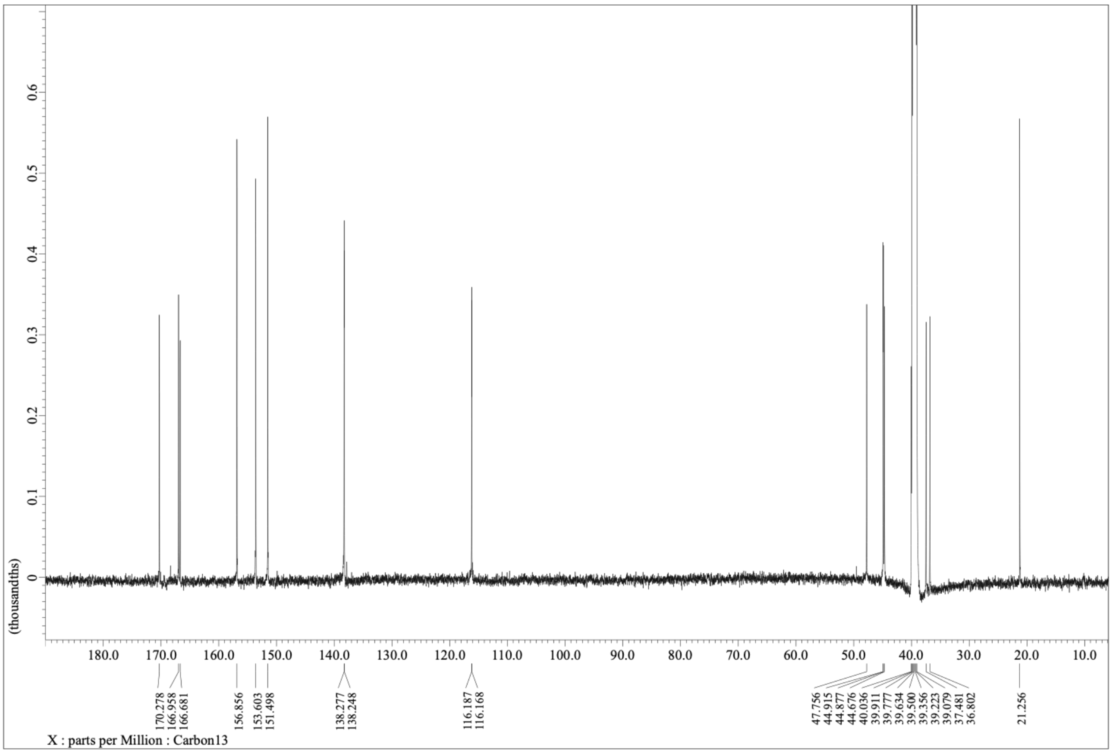

^{3}C NMR spectrum of **12**

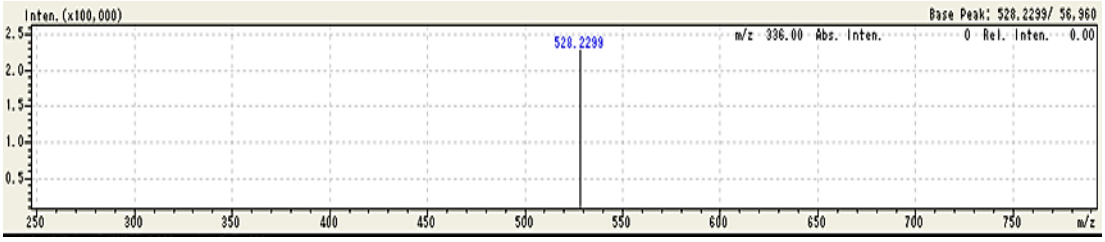

ESI-MS chromatogram of **12**

_N,N_-Bis(2-(2-(2-amino-6-oxo-1_H_-purin-9(6_H_)-yl)acetamido)ethyl) propionamide (13)

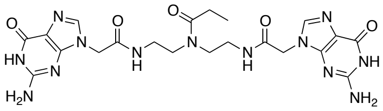

Compound **10** (40 mg, 0.06 mmol) was dissolved in CH_2Cl_2 (600 µL) and DIPEA (52.6 µL, 5 eq), then stirred at 0ºC. Propionyl chloride (10.5 µL, 2 eq) was added into the mixture, stirred for 2 h at rt. DIPEA (1 eq) and propionyl chloride (1 eq) was added into the mixture, stirred for 1 h at rt. The reaction mixture was concentrated under reduced pressure, dried under vacuum. The residue was added NH_3 in MeOH (7 M, 5 mL), stirred for over weekend at rt. The reaction mixture was concentrated under reduced pressure. The residue was added NH_3 in MeOH (7 M, 5 mL), stirred over weekend at rt. The reaction mixture was concentrated under reduced pressure. The residue was added MeOH, the precipitate was collected by centrifugation, washed with MeOH, dried under vacuum to afford **13** (9.7 mg, 0.018 mmol, 30% yield for 2 steps) as a pale yellow solid.

^{1}H NMR (600 MHz, DMSO-d_6) δ 8.23 (dt, J = 43.2, 5.9 Hz, 2H), 7.58 (d, J = 2.1 Hz, 2H), 6.42 (s, 4H), 4.59 (d, J = 14.5 Hz, 4H), 3.29-3.15 (m, 5H), 2.27 (q, J = 7.3 Hz, 2H), 0.95 (t, J = 7.2 Hz, 3H); ^{13}C NMR (151 MHz, DMSO-d_6) δ 173.2, 166.9, 166.7, 156.9, 153.6, 153.6, 151.5, 138.3, 138.2, 116.2, 46.8, 45.0, 44.9, 40.0, 39.9, 39.8, 39.6, 39.5, 39.4, 39.2, 39.1, 38.8, 37.6, 36.8, 25.3, 9.5. ESI-HRMS calcd for $C_{21}H_{28}N_{13}O_5$ $[M+2H]^{2+}$: 271.6202, found: 271.6240.

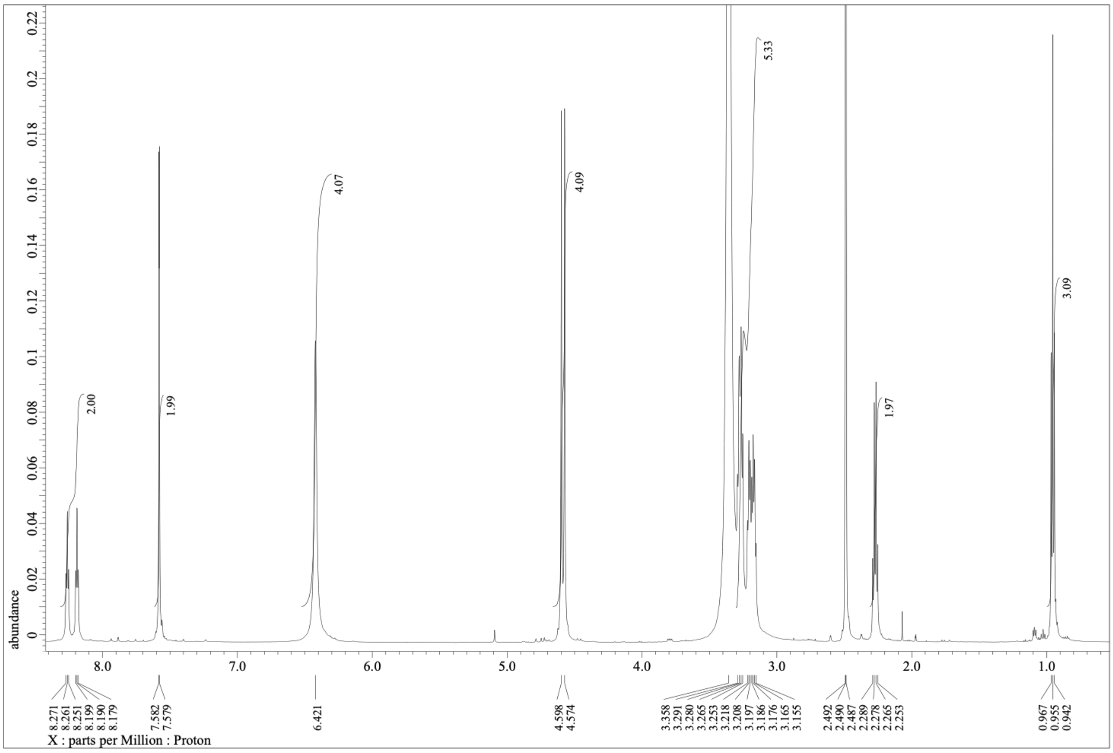

^{1}H NMR spectrum of **13**

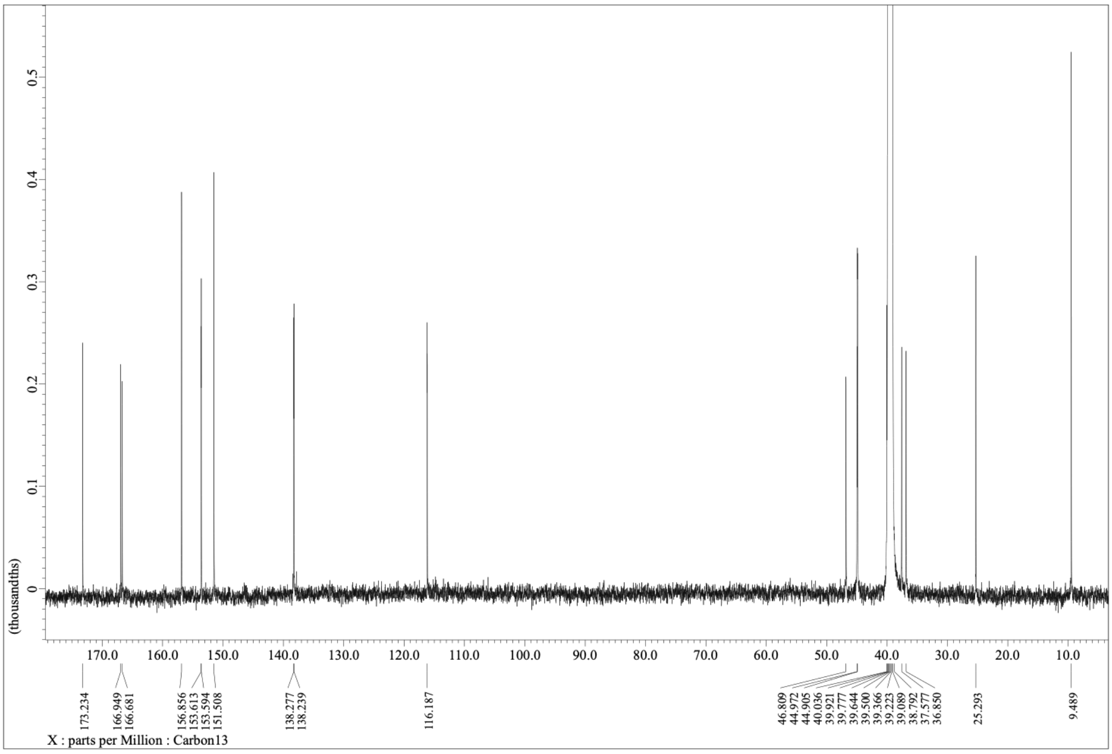

^{13}C NMR spectrum of **13**

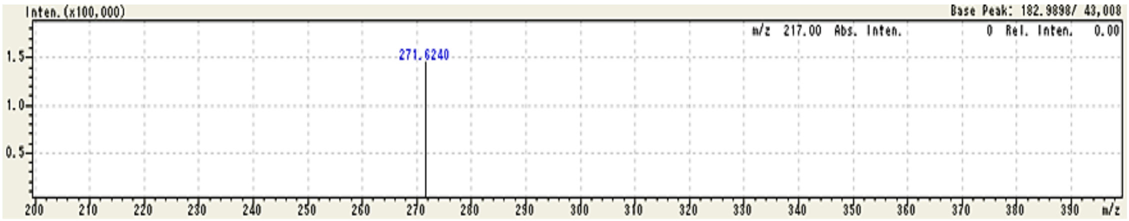

ESI-MS chromatogram of **13**

***N*,*N*-Bis(2-(2-(2-amino-6-oxo-1*H*-purin-9(6*H*)-yl)acetamido)ethyl) isobutyramide (14)**

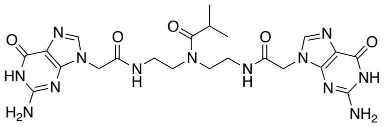

Compound **10** (40 mg, 0.06 mmol) was dissolved in CH_2Cl_2 (600 µL) and DIPEA (52.6 µL, 5 eq), then stirred at 0ºC. Isobutyryl chloride (13 µL, 2 eq) was added into the mixture, stirred for 2 h at rt. DIPEA (1 eq) and isobutyryl chloride (1 eq) was added into the mixture, stirred for 1 h at rt. The reaction mixture was concentrated under reduced pressure, dried under vacuum. The residue was added NH_3 in MeOH (7 M, 5 mL), stirred over weekend at rt. The reaction mixture was concentrated under reduced pressure. The residue was added MeOH, the precipitate was collected by centrifugation, washed with MeOH, dried under vacuum to afford **14** (8.5 mg, 0.015 mmol, 26% yield for 2 steps) as a pale yellow solid.

^{1}H NMR (600 MHz, DMSO-*d*$_6$) δ 8.24 (dt, *J* = 59.2, 5.9 Hz, 2H), 7.58 (d, *J* = 9.6 Hz, 2H), 6.42 (s, 4H), 4.58 (d, *J* = 15.8 Hz, 4H), 3.32-3.15 (m, 4H), 2.78 (sept., *J* = 6.7 Hz, 1H), 0.96 (d, *J* = 6.9 Hz, 6H); ^{13}C NMR (151 MHz, DMSO-*d*$_6$) δ 176.7, 167.0, 166.7, 156.9, 153.6, 151.5, 138.3, 138.3, 116.2, 46.6, 45.0, 44.9, 40.0, 38.1, 36.9, 29.1, 19.7. ESI-HRMS calcd for $C_{22}H_{30}N_{13}O_5$ $[M+H]^+$: 556.2487, found: 566.2526.

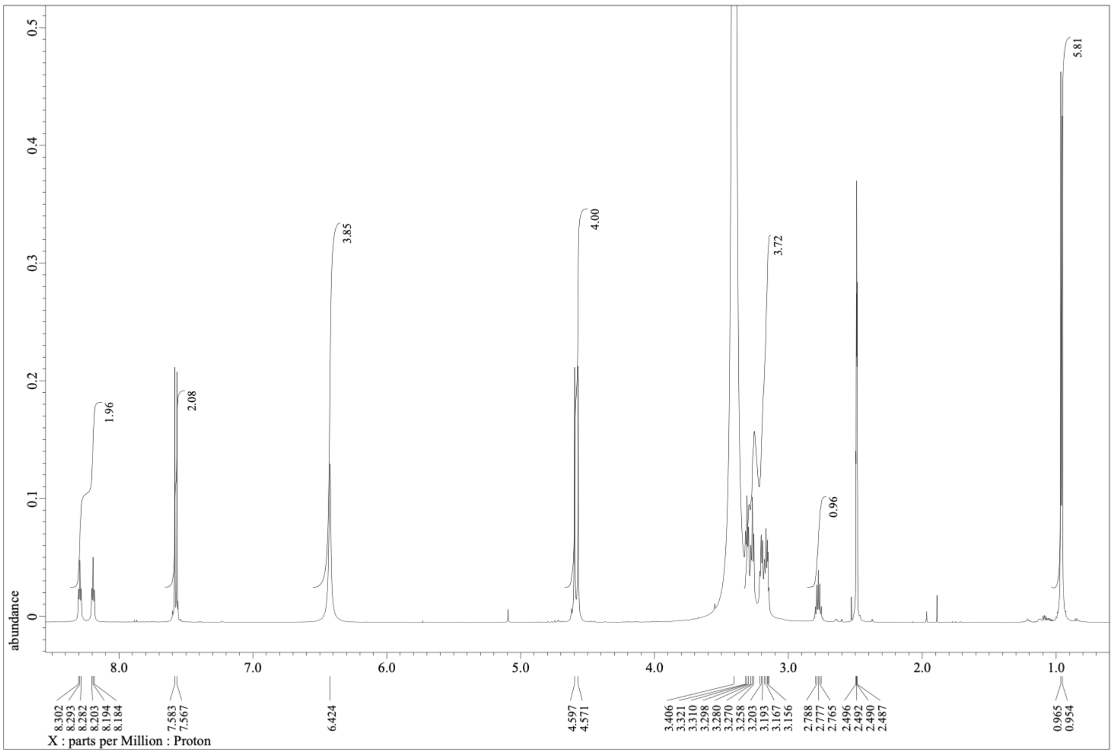

^{1}H NMR spectrum of **14**

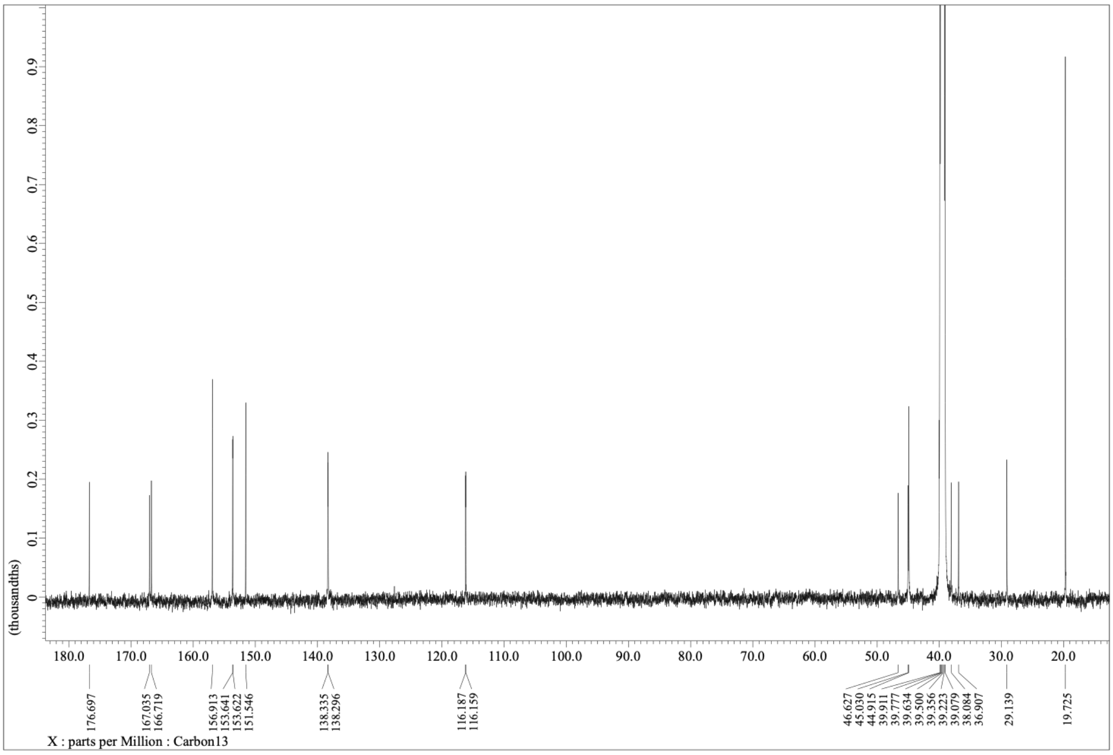

^{13}C NMR spectrum of **14**

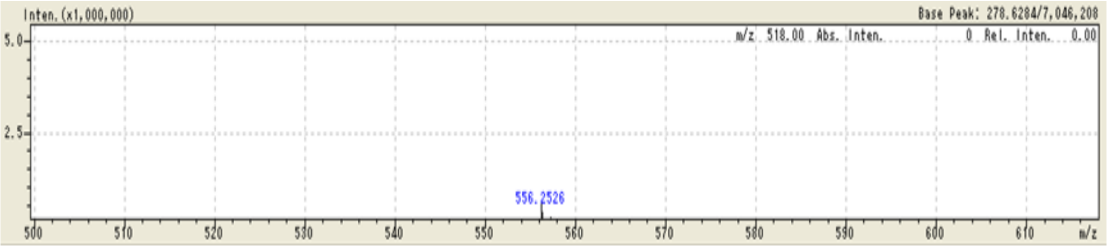

ESI-MS chromatogram of **14**

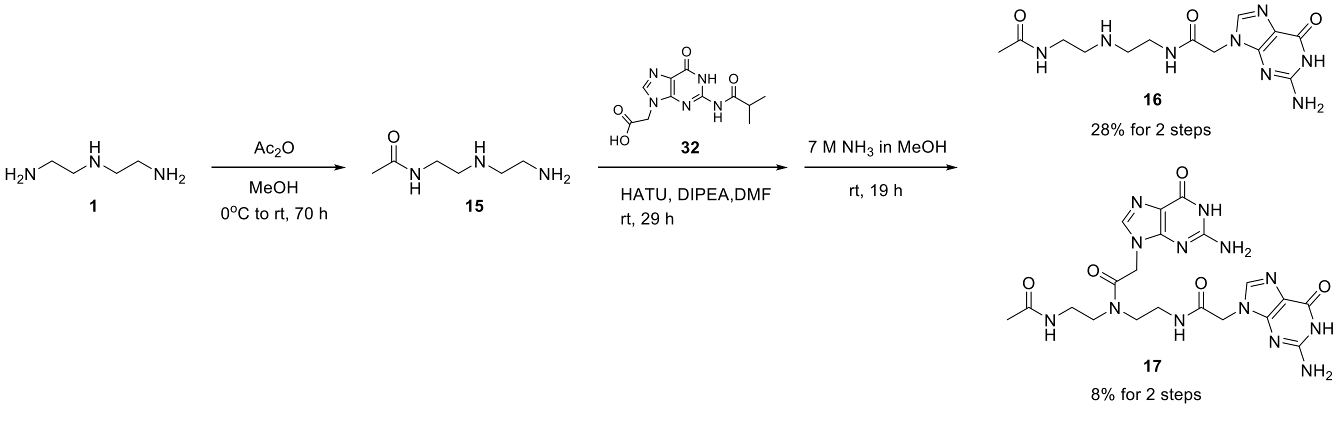

Scheme S4. Synthesis of compounds **16** and **17**

***N*-(2-((2-Aminoethyl)amino)ethyl)acetamide (15)**

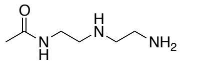

Compound **1** (1 mL, 0.01 mmol) was dissolved in MeOH (100 mL), stirred at 0ºC. Acetic anhydride (5.7 mL, 0.2 eq) dissolved in MeOH (100 mL) was added dropwise over 4.5 h using a dropping funnel, stirred over weekend at 0ºC to rt. The reaction mixture was concentrated under reduced pressure to remove solvent, diluted with CH_2Cl_2, extracted with H_2O. The aqueous layer was concentrated under reduced pressure, dried under vacuum. The residue was purified by flash silica gel column chromatography (φ = 4 cm, h = 13 cm, CH_2Cl_2/MeOH/28% NH_4OH = 20:5:1 to 18:7:1), followed by purified by flash silica gel column chromatography (φ = 4 cm, h = 11 cm, CH_2Cl_2/MeOH/28% NH_4OH = 60:30:0 to 57:33:0 to 57:33:10) to afford **15** (1.14 g, 7.85 mmol, 79% yield) as a pale yellow oil.

^{1}H NMR (600 MHz, DMSO-*d*6) δ 8.26 (s, 1H), 3.50 (q, *J* = 6.2 Hz, 2H), 2.97 (t, *J* = 5.9 Hz, 2H), 2.93 (t, *J* = 6.5 Hz, 2H), 2.89 (t, *J* = 6.2 Hz, 2H), 2.20 (s, 3H).

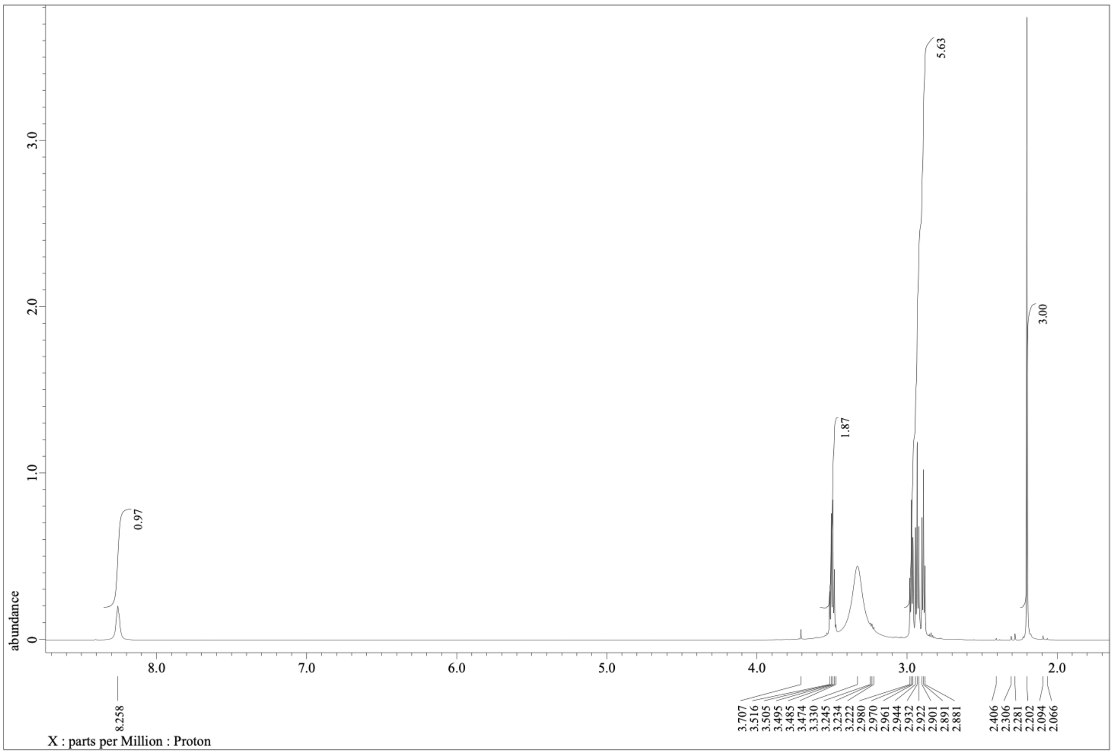

^{1}H NMR spectrum of **15**

***N*-(2-((2-Acetamidoethyl)amino)ethyl)-2-(2-amino-6-oxo-1*H*-purin-9(6*H*)-yl)acetamide (16) and *N*-(2-Acetamidoethyl)-2-(2-amino-6-oxo-1*H*-purin-9(6*H*)-yl)-*N*-(2-(2-(2-amino-6-oxo-1*H*-purin-9(6*H*)-yl)acetamido)ethyl)acetamide (17)**

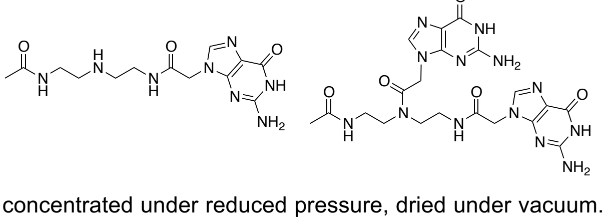

Compound **15** (145 mg, 1 mmol) was dissolved in DMF (5 mL) and stirred at 0ºC. Compound **32** (279 mg, 1 eq), HATU (570 mg, 1.5 eq) and DIPEA (523 µL, 3 eq) was added into the mixture, stirred overnight at rt. The reaction mixture was diluted with EtOAc, extracted with H_2O. The aqueous layer was concentrated under reduced pressure, dried codistillation with MeOH. A half of the residue was added NH_3 in MeOH (7 M, 15 mL), stirred over weekend at rt. The reaction mixture was concentrated under reduced pressure, dried under vacuum. The residue was purified by flash silica gel column chromatography (φ = 2.5 cm, h = 10.5 cm, CH_2Cl_2/MeOH/28% NH_4OH = 85:15:0 to 40:50:10) to afford **16** (47 mg, 0.14 mmol, 28% yield for 2 steps) as a pale orange solid and **17** (20 mg, 0.038 mmol, 8% yield for 2 steps) as a pale orange solid.

For **16** : ^{1}H NMR (600 MHz, DMSO-*d*6) δ 8.23 (t, *J* = 5.2 Hz, 1H), 7.89 (d, *J* = 4.8 Hz, 1H), 7.60 (s, 1H), 6.43 (s, 2H), 4.62 (s, 2H), 3.14 (dq, *J* = 30.5, 6.0 Hz, 4H), 2.65-2.61 (dt, *J* = 13.6 Hz, 6 Hz, 3H), 1.79 (s, 3H); ^{13}C NMR (151 MHz, DMSO-*d*6) δ 169.7, 166.7, 157.0, 153.6, 151.6, 138.5, 116.1, 48.0, 47.8, 44.9, 38.3, 38.1, 22.7. ESI-HRMS calcd for $C_{13}H_{21}N_8O_3$ $[M+H]^+$: 337.1731, found: 337.1850.

For **17** : ^{1}H NMR (600 MHz, DMSO-*d*6) δ 8.35 (t, *J* = 5.5 Hz, 1H), 8.21 (t, *J* = 5.9 Hz, 1H), 8.08 (t, *J* = 5.9 Hz, 1H), 7.89 (t, *J* = 5.9 Hz, 1H), 7.60 (s, 1H), 7.56 (d, *J* = 1.4 Hz, 2H), 7.54 (s, 1H), 6.43-6.35 (m, 10H), 4.88 (s, 4H), 4.70 (s, 2H), 4.59 (s, 2H), 3.41 (q, *J* = 6.9 Hz, 4H), 3.17 (dq, *J* = 34.1, 6.5 Hz, 4H), 1.85 (s, 3H), 1.76 (s, 3H); ^{13}C NMR (151 MHz, DMSO-*d*6) δ 170.0, 169.5, 167.2, 166.7, 166.7, 166.6, 156.8, 153.6, 153.5, 151.6, 151.5, 138.5, 138.4, 138.3, 138.2, 116.2, 116.1, 116.0, 46.3, 46.0, 45.4, 45.3, 45.0, 44.8, 43.6, 43.5,

40.1, 37.4, 37.3, 36.5, 36.3, 22.7, 22.6. ESI-HRMS calcd for $C_{20}H_{26}N_{13}O_5$ $[M+H]^+$: 528.2174, found: 528.2177.

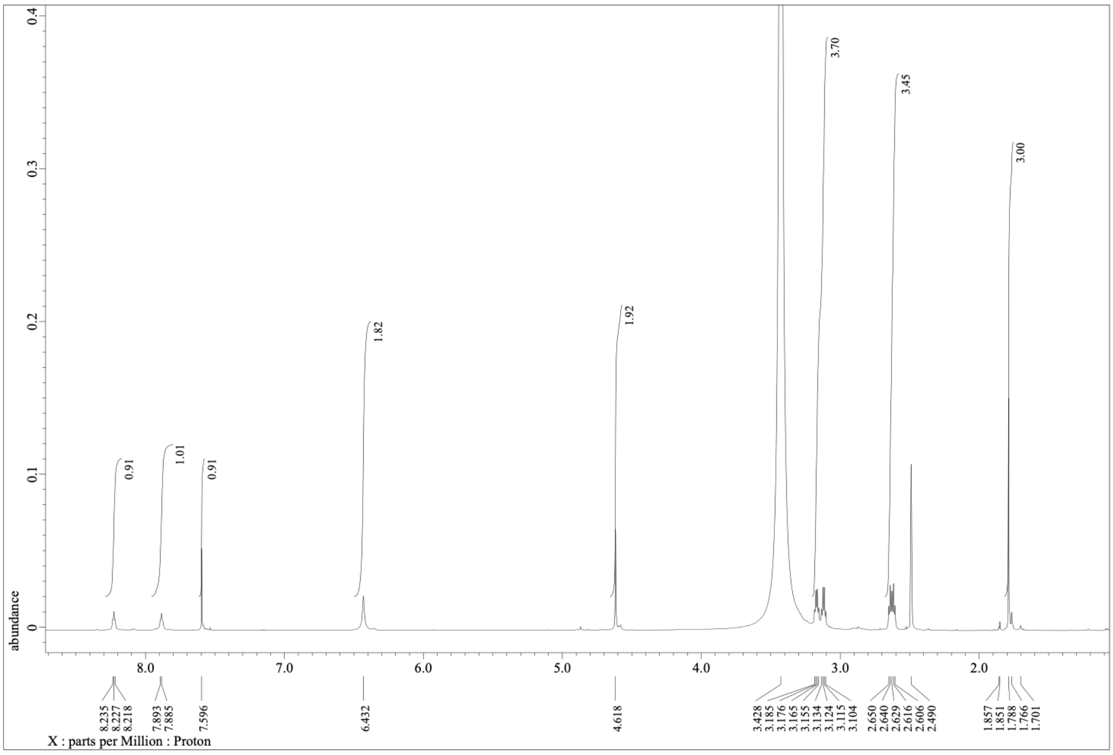

^1H NMR spectrum of **16**

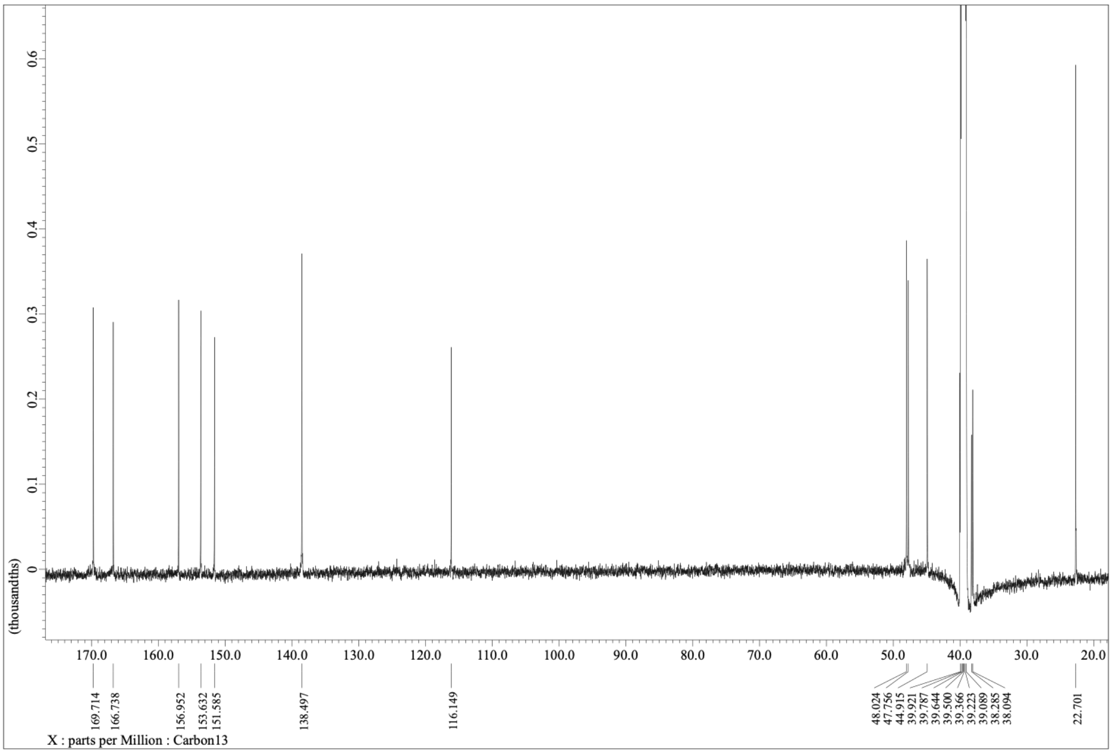

^{13}C NMR spectrum of **16**

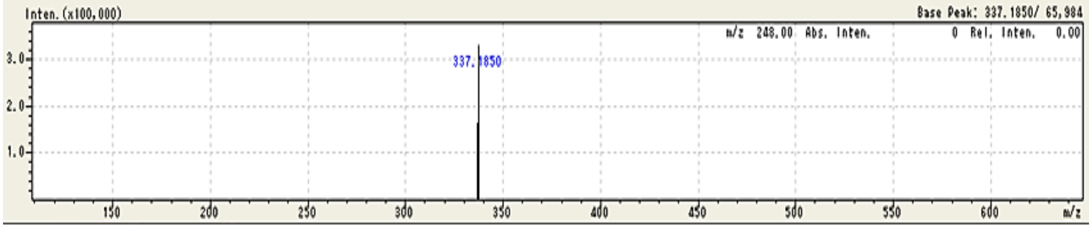

ESI-MS chromatogram of **16**

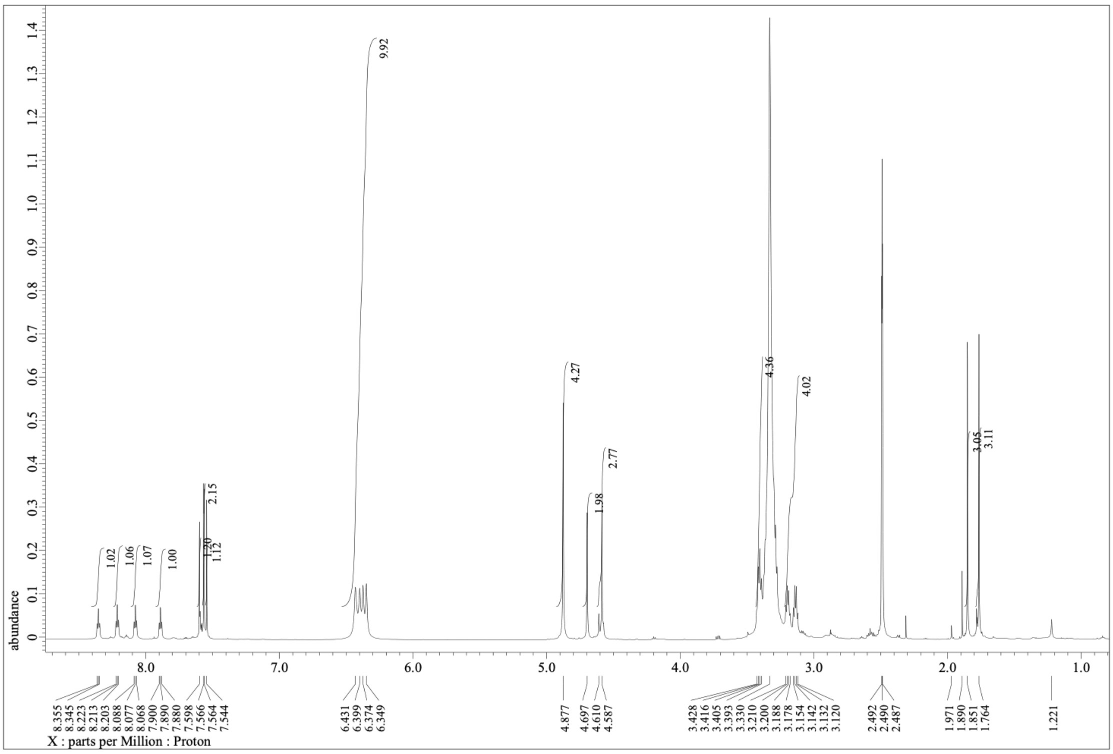

^{1}H NMR spectrum of **17**

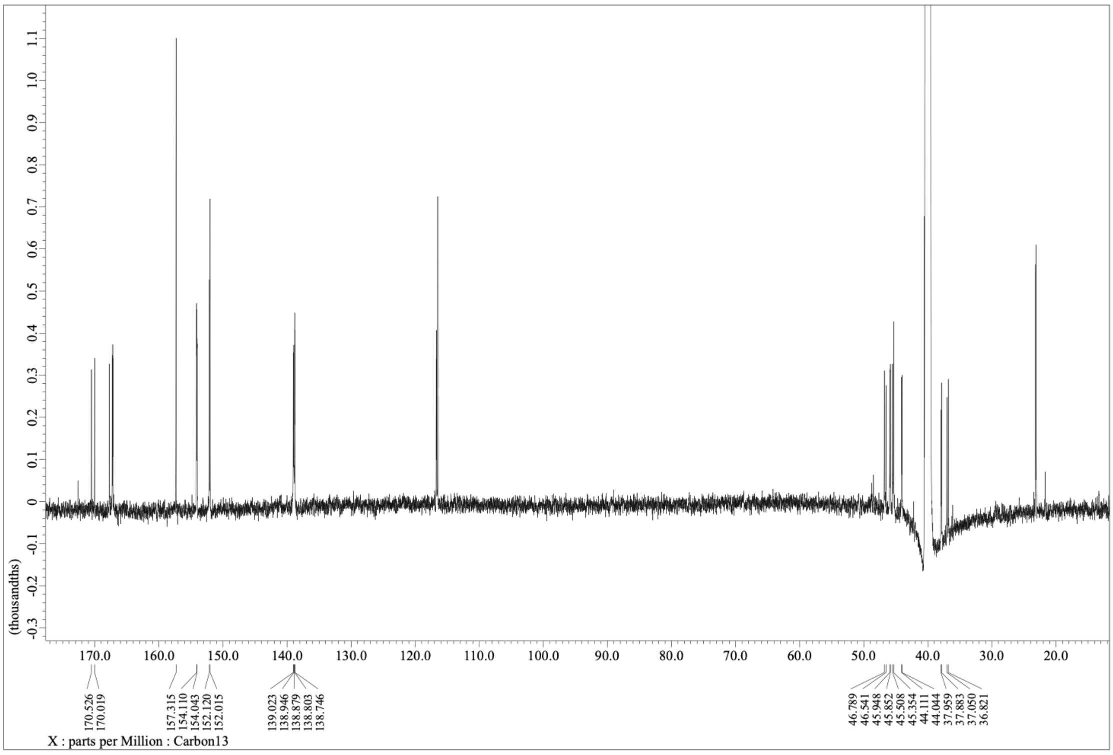

^{13}C NMR spectrum of **17**

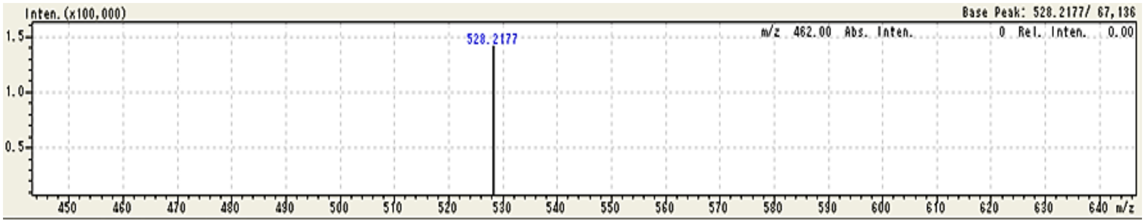

ESI-MS chromatogram of **17**

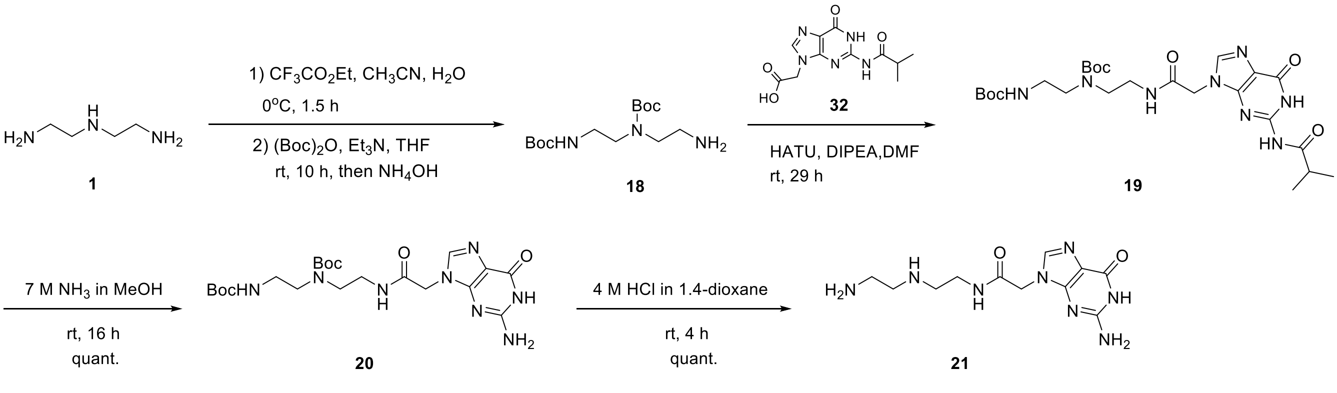

Scheme S5. Synthesis of compound **21**

tert-Butyl (2-aminoethyl)(2-((_tert_-butoxycarbonyl)amino)ethyl)carbamate (18)

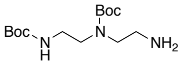

Compound **1** (1.03 g, 10 mmol) was dissolved in MeOH (40 mL), stirred at -78ºC. Ethyl trifluoroacetate (1.25 mL, 1.05eq) in MeOH (50 mL) was added dropwise into the mixture over 30 min using a dropping funnel, then stirred 1.5 h at 0ºC. $(Boc)_2O$ (6.89 g, 3 eq) in MeOH (10 mL) was added into the mixture, stirred for 10 h at rt. The reaction mixture was concentrated under reduced pressure to give a colorless oil. The oil was dissolved in MeOH (15 mL), added 28% NH_4OH (15 mL) at rt, stirred for 24 h at 50ºC. After cooling to rt, the reaction mixture was concentrated under reduced pressure to remove the volatiles. The residue was added 1 M NaOH aq., and extracted with CH_2Cl_2 (100 mL) 3 times. The combined organic extracts were dried over Na_2SO_4, filtered, concentrated under reduced pressure. Purified by flash silica gel column chromatography (φ = 2.5 cm, h = 15 cm, CH_2Cl_2/MeOH/28% NH_4OH = 100:5:1 to 100:10:1) to afford **18** (1.82 g, 6.0 mmol, 60% yield) as a white solid.

^{1}H NMR (600 MHz, $CDCl_3$) δ 5.41 (d, J = 123.3 Hz, 1H), 3.33-3.28 (m, 6H), 2.85 (br.s, 2H), 1.47 (s, 9H), 1.43 (s, 9H); ^{13}C NMR (151 MHz, $CDCl_3$) δ 156.0, 155.9, 80.0, 79.1, 51.1, 50.6, 47.7, 47.2, 40.9, 40.4, 39.6, 28.4. ESI-HRMS calcd for $C_{14}H_{30}N_3O_4$ $[M+H]^+$: 304.2231, found: 304.2239.

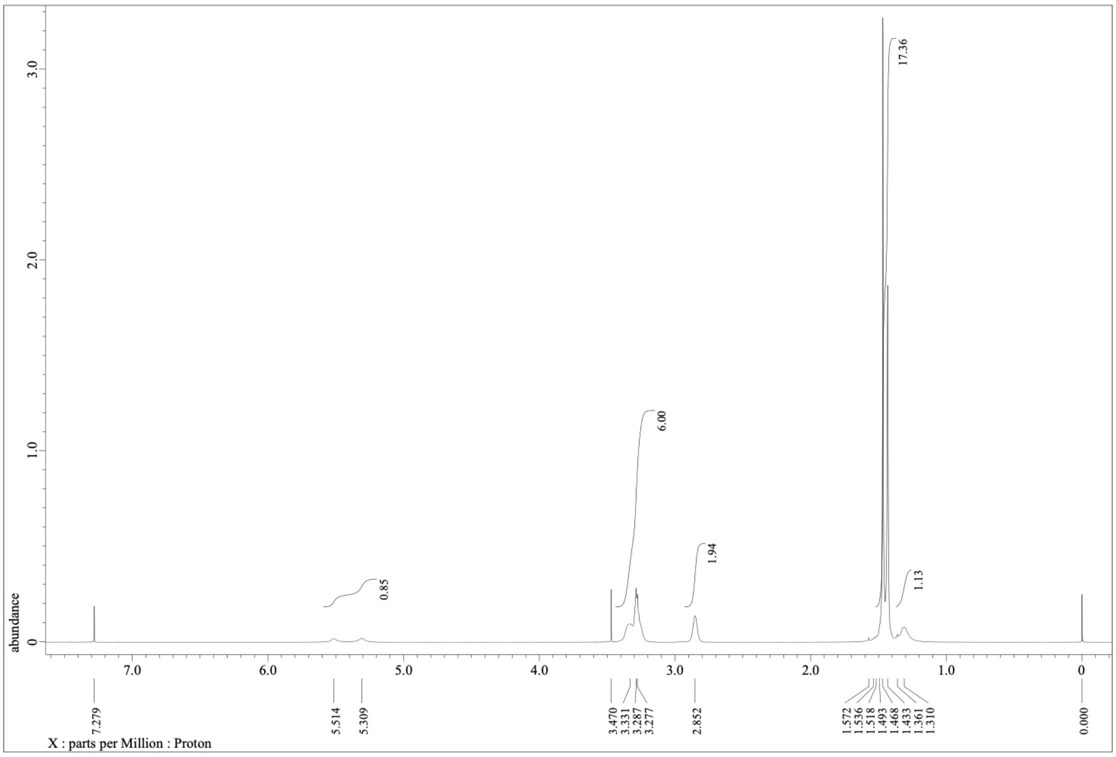

^{1}H NMR spectrum of **18**

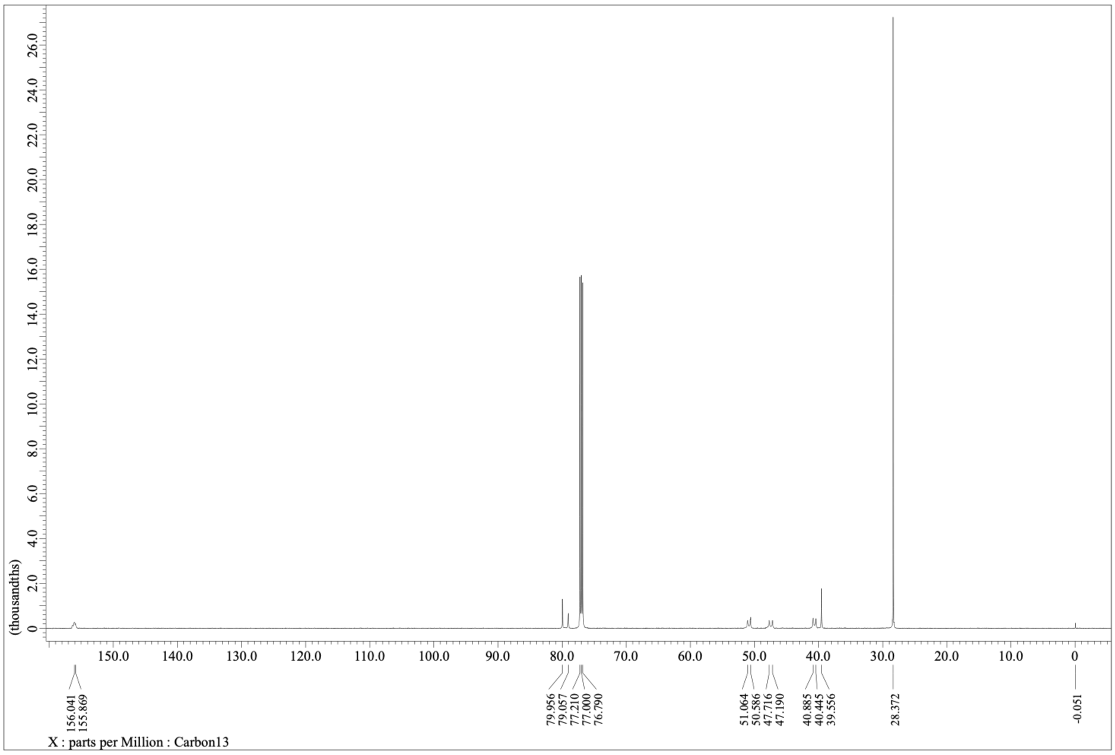

^{13}C NMR spectrum of **18**

tert-Butyl (2-((_tert_-butoxycarbonyl)amino)ethyl)(2-(2-(2-isobutyramido-6-oxo-1_H_-purin-9(6_H_)-yl)acetamido)ethyl)carbamate (19)

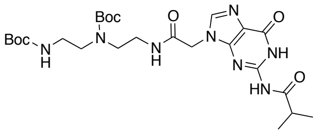

Compound **18** (30 mg, 0.1 mmol) was dissolved in DMF (500 µL) and stirred at 0ºC. Compound **32** (31 mg, 1.1 eq), HATU (57 mg, 1.5 eq) and DIPEA (52 µL, 3 eq) was added into the mixture, stirred over weekend at rt. The reaction mixture was diluted with EtOAc, washed with HCl (1 M), brine. The aqueous layer was extracted with CH_2Cl_2 3 times. The organic layer was dried over Na_2SO_4, filtered, concentrated under reduced pressure. The residue was purified by flash NH silica gel column chromatography (*φ* = 1.5 cm, h = 10 cm, CH_2Cl_2/MeOH = 100:0 to 85:15) to afford **19** (24 mg, 0.043 mmol, 43% yield) as a pale yellow solid.

^{1}H NMR (600 MHz, $CDCl_3$) δ 7.70 (d, *J* = 17.9 Hz, 1H), 5.43-5.21 (m, 1H), 4.84-4.73 (m, 2H), 3.50-3.29 (m, 8H), 3.03-2.96 (m, 2H), 2.89-2.85 (m, 1H), 1.49-1.41 (m, 18H), 1.25 (s, *J* = 6.2 Hz, 6H). ESI-HRMS calcd for $C_{25}H_{41}N_8O_7$ $[M+H]^+$: 565.3093, found: 565.3116.

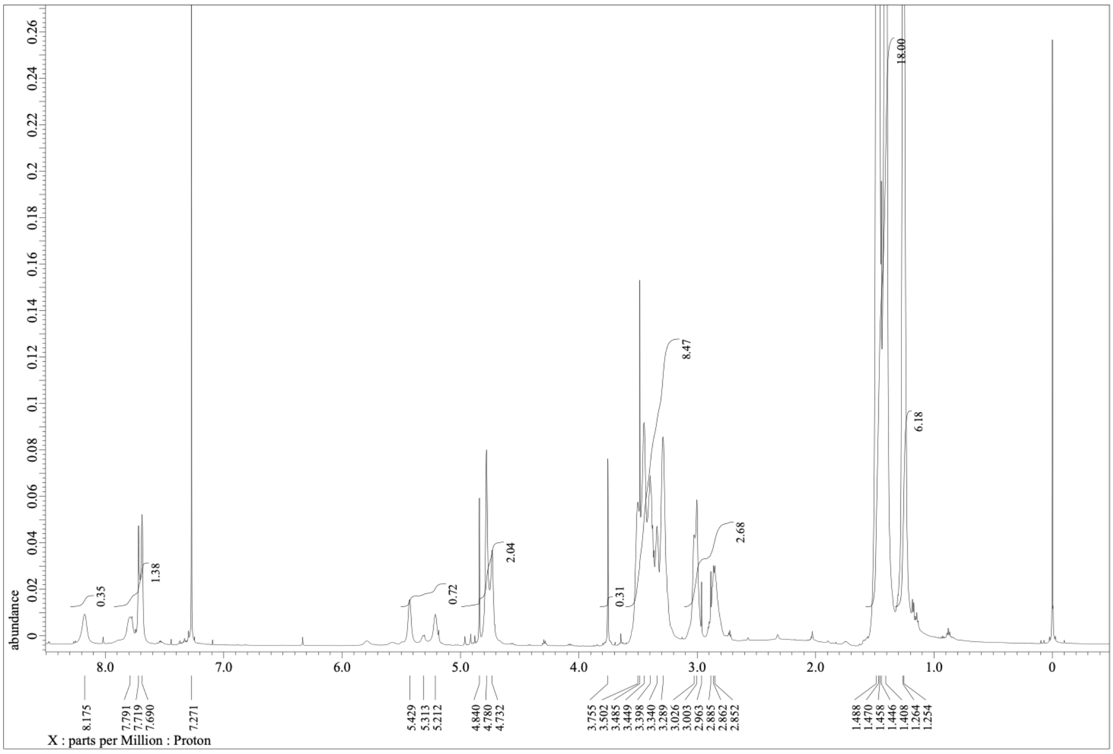

^1H NMR spectrum of **19**

tert-Butyl (2-(2-(2-amino-6-oxo-1_H_-purin-9(6_H_)-yl)acetamido)ethyl)(2-((_tert_-butoxycarbonyl)amino)ethyl)carbamate (20)

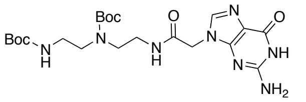

Compound **19** (24 mg, 0.043 mmol) was added NH_3 in MeOH (7 M, 10 mL), stirred for 18 h at rt. The reaction mixture was concentrated under reduced pressure, dried under vacuum to afford **20** (24 mg, 0.049 mmol, quant.) as a pale yellow solid.

^1H NMR (600 MHz, $CDCl_3$) δ 4.71 (br.s, 1H), 3.48-3.22 (m, 7H), 2.96-2.88 (m, 2H), 1.46-1.25 (m, 18H). ESI-HRMS calcd for $C_{21}H_{35}N_8O_6$ $[M+H]^+$: 495.2674, found: 495.2709.

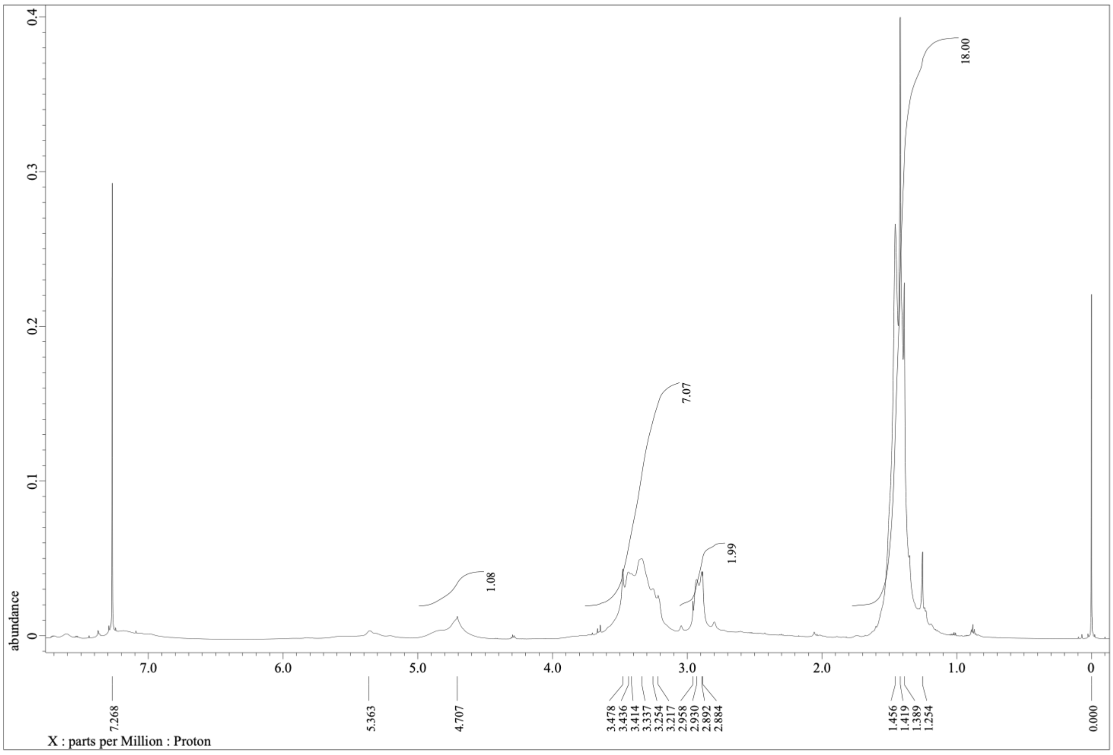

^{1}H NMR spectrum of **20**

2-(2-Amino-6-oxo-1*H*-purin-9(6*H*)-yl)-*N*-(2-((2-aminoethyl)amino)ethyl) acetamide (21)

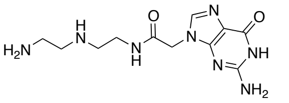

Compound **20** (24 mg, 0.049 mmol) was added HCl in 1,4-dioxane (4 M, 10 mL), stirred for 1 h at rt. The reaction mixture was concentrated under reduced pressure. The residue was added ACN, the precipitate was collected by centrifugation, washed with ACN 2 times, dried under vacuum to afford **21** (15.5 mg, 0.053 mmol, quant.) as a white solid.

^{1}H NMR (600 MHz, CD_3OD) δ 9.19 (s, 1H), 5.12 (s, 2H), 3.74-3.57 (m, 4H), 3.41 (dt, *J* = 13.3, 5.2 Hz, 4H), 3.03 (s, 1H); ^{13}C NMR (151 MHz, CD_3OD) δ 168.6, 157.4, 155.1, 152.0, 139.5, 108.4, 73.5, 72.4, 62.2, 47.5, 45.9, 43.8, 40.6, 37.2, 37.0. ESI-HRMS calcd for $C_{11}H_{19}N_8O_2$ $[M+H]^+$: 295.1625, found: 294.9645.

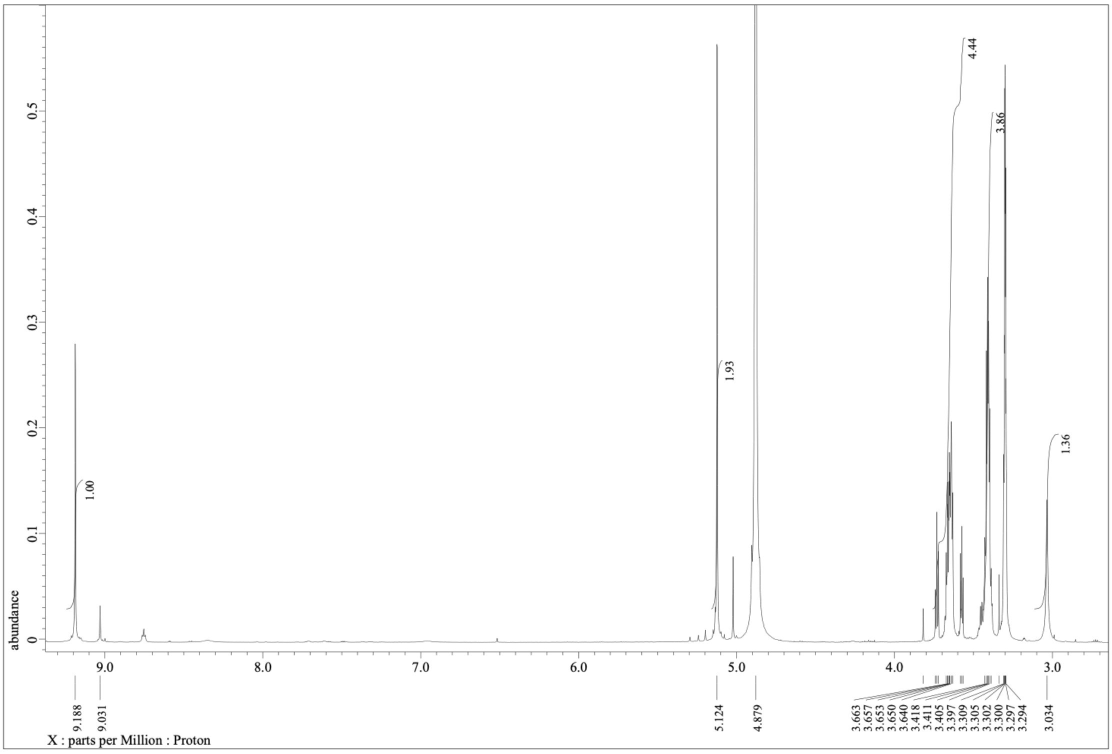

^{1}H NMR spectrum of **21**

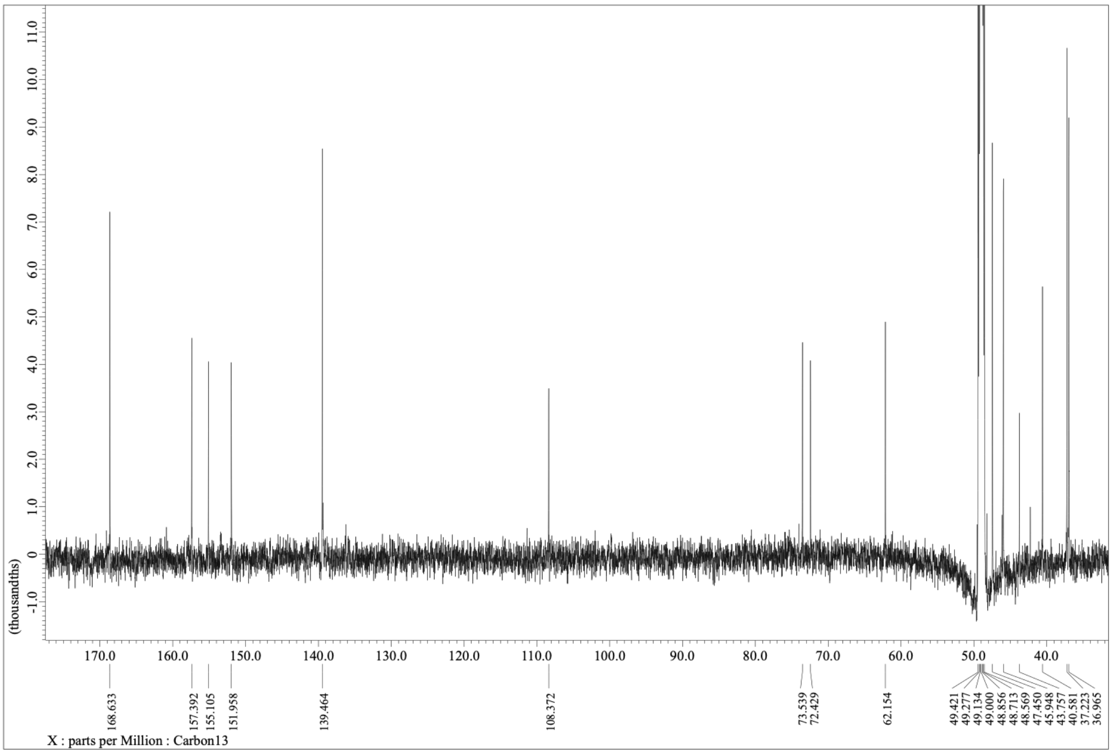

^{13}C NMR spectrum of **21**

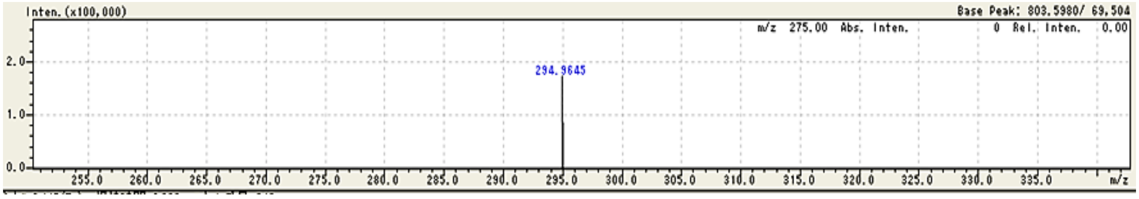

ESI-MS chromatogram of **21**

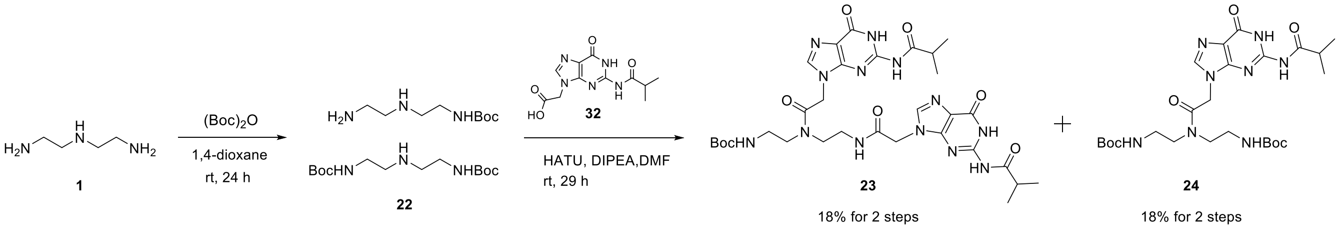

Scheme S7. Synthesis of compounds **23** and **24**

***tert*-Butyl (2-((2-aminoethyl)amino)ethyl)carbamate (22)[S3]**

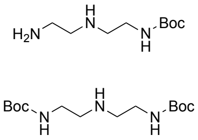

Compound **1** (10.9 mL, 100 mmol) was dissolved in 1,4-dioxane (17 mL), stirred at rt. $(Boc)_2O$ (3.1 g, 0.14 eq) in 1,4-dioxane (20 mL) was added dropwise over 1 h using a dropping funnel, stirred for 24 h. The reaction mixture was concentrated under reduced pressure at 50ºC. The residue was added H_2O (15 mL) then filtered. The filtrate was extracted with CH_2Cl_2 (9 mL) 5 times. The combined extracts were dried over Na_2SO_4, filtered, concentrated under reduced pressure, dried under vacuum to afford mixture **22** (1.98 g) as a colorless oil.

^{1}H NMR (600 MHz, $CDCl_3$) δ 5.00 (br.s, 1H), 3.22 (s, 2H), 2.85-2.66 (m, 6H), 1.45 (s, 9H). ESI-HRMS calcd for $C_9H_{22}N_3O_2$ $[M+H]^+$: 204.1707, found: 204.1698.

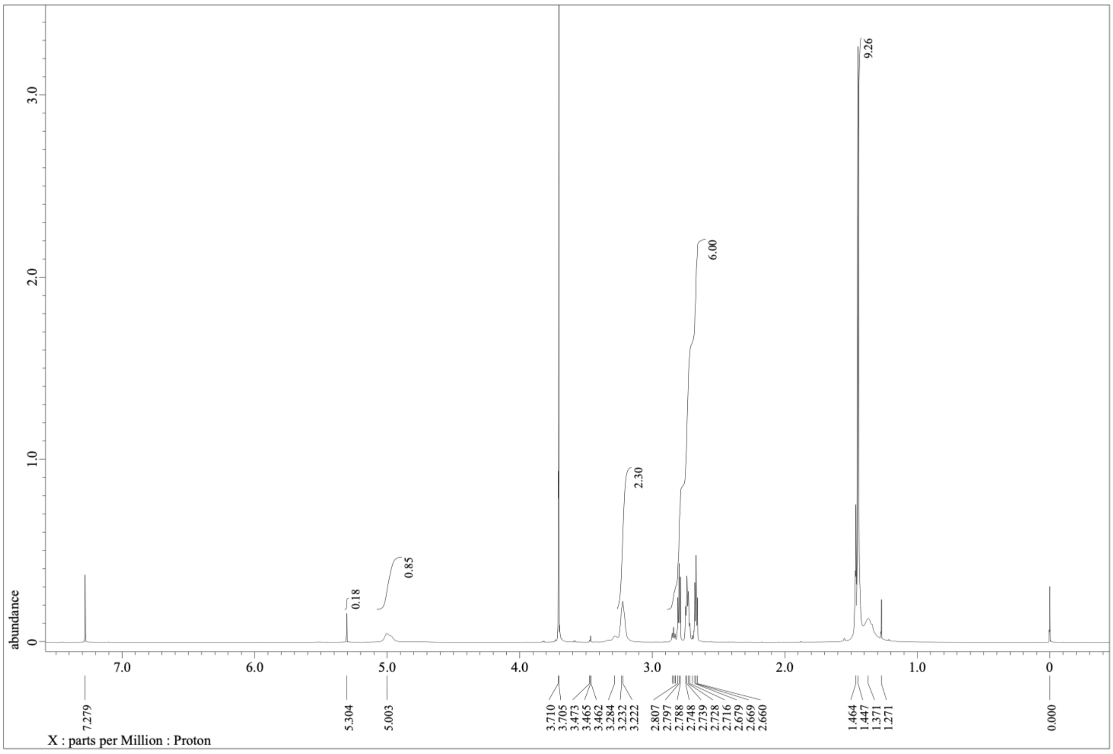

^{1}H NMR spectrum of **22**

***tert*-Butyl (2-(2-(2-isobutyramido-6-oxo-1*H*-purin-9(6*H*)-yl)-*N*-(2-(2-(2-isobutyramido-6-oxo-1*H*-purin-9(6*H*)-yl)acetamido)ethyl)acetamido)ethyl) carbamate (23) and Di-*tert*-butyl (((2-(2-isobutyramido-6-oxo-1*H*-purin-9(6*H*)-yl)acetyl) azanediyl)bis(ethane-2,1-diyl))dicarbamate (24)**

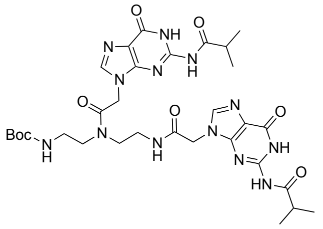

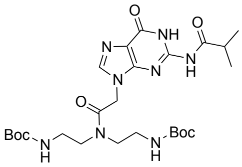

Mixture **22** (102 mg) was dissolved in DMF (2.5 mL) and stirred at 0ºC. Compound **32** (305 mg, 2.2 eq), HATU (570 mg, 3 eq) and DIPEA (523 µL, 6 eq) was added into the mixture, stirred for 29 h at rt. The reaction mixture was diluted with EtOAc, washed with HCl (1 M), brine. The organic layer was dried over Na_2SO_4, filtered, concentrated under reduced pressure, dried under vacuum. The residue was purified by flash silica gel column chromatography (Yamazen inject column size: M, Yamazen Hi-flash column size: M, CH_2Cl_2/MeOH = 99:1 to 80:20) to afford **23** (91 mg, 0.13 mmol, 18% yield for 2 steps) as a pale orange solid and **24** (72 mg, 0.13 mmol, 18% for 2 steps) as a dull orange gum.

For **23** : ^{1}H NMR (600 MHz, CD_3OD) δ 8.63 (d, *J* = 4.1 Hz, 2H), 8.34-8.32 (d, *J* = 7.2 Hz, 2H), 7.44 (q, *J* = 4.4 Hz, 2H), 7.20 (t, *J* = 7.6 Hz, 1H), 7.15-7.08 (m, 1H), 5.15 (t, *J* = 23.1 Hz, 2H), 3.74-3.34 (m, 1H), 3.26-3.18 (m, 1H), 2.74-2.61 (m, 2H), 2.31 (s, 1H), 1.40 (d, *J* = 4.1 Hz, 8H), 1.37-1.34 (m, 6H), 1.19-1.18 (m, 9H). ESI-HRMS calcd for $C_{31}H_{44}N_{13}O_8$ [M+H]$^+$: 726.3430, found: 726.3393.

For **24** : ^{1}H NMR (600 MHz, CD_3OD) δ 8.68 (t, *J* = 2.4 Hz, 1H), 8.38 (dd, *J* = 8.3, 1.4 Hz, 1H), 7.97-7.90 (m, 1H), 7.48 (q, *J* = 4.4 Hz, 1H), 7.20 (t, *J* = 7.6 Hz, 2H), 7.14 (d, *J* = 6.9 Hz, 2H), 7.10 (t, *J* = 7.2 Hz, 1H), 5.13 (s, 1H), 3.72 (sept., *J* = 6.5 Hz, 3H), 3.56 (t, *J* = 6.2 Hz, 1H), 3.44 (t, *J* = 6.2 Hz, 1H), 3.36 (t, *J* = 6.5 Hz, 1H), 3.22 (q, *J* = 7.3 Hz, 4H), 2.73-2.68 (m, 1H), 2.31 (s, 3H), 1.41 (s, 10H), 1.37-1.34 (m, 25H), 1.22-1.19 (m, 6H). ESI-HRMS calcd for $C_{25}H_{41}N_8O_7$ [M+H]$^+$: 565.3093, found: 565.3061.

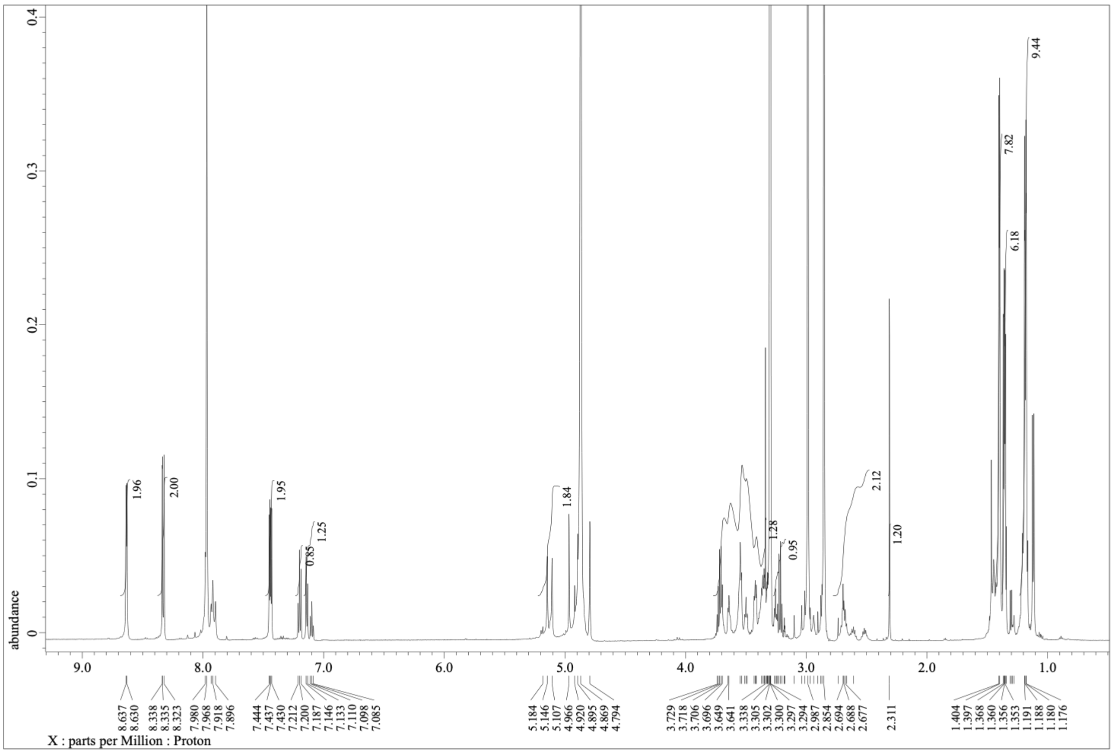

^{1}H NMR spectrum of **23**

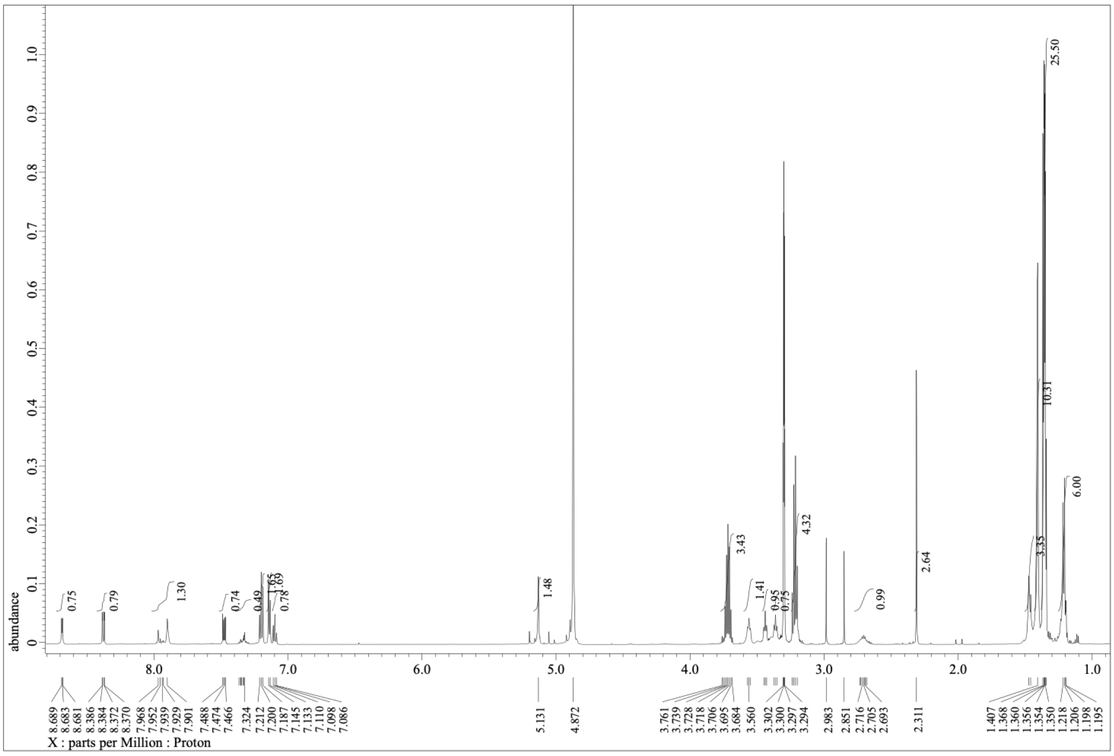

^{1}H NMR spectrum of **24**

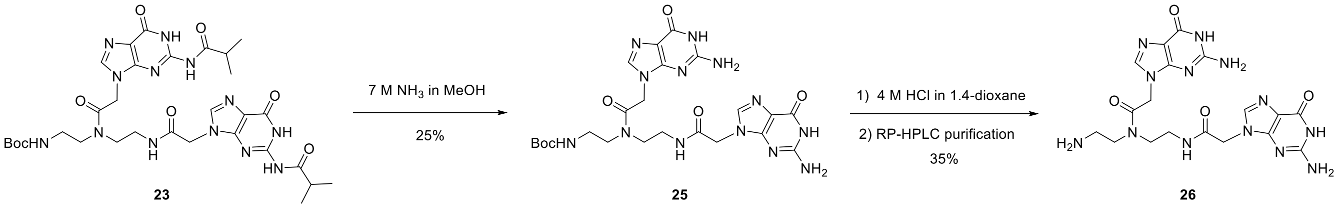

Scheme S8. Synthesis of compound **26**

***tert*-Butyl (2-(2-(2-amino-6-oxo-1*H*-purin-9(6*H*)-yl)-*N*-(2-(2-(2-amino-6-oxo-1*H*-purin-9(6*H*)-yl)acetamido)ethyl)acetamido)ethyl)carbamate (25)**

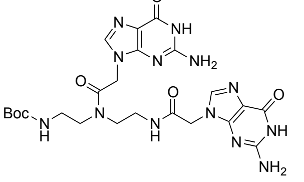

Compound **23** (91 mg, 0.13 mmol) was added NH_3 in MeOH (7 M, 5 mL), stirred for 20 h at rt. The reaction mixture was concentrated under reduced pressure. The residue was added NH_3 in MeOH (7 M, 5 mL), stirred for 23 h at 30ºC. The resulting precipitate was collected by centrifugation, washed with ACN 2 times, dried under vacuum to afford **25** (18.6 mg, 0.032 mmol, 25% yield) as a pale orange solid. ^{1}H NMR (600 MHz, DMSO-*d*$_6$) δ 7.59-7.52 (m, 2H), 6.40 (br.s, 1H), 6.35 (br.s, 2H), 4.86 (d, *J* = 4.1 Hz, 2H), 4.69 (s, 1H), 4.58 (s, 1H), 3.20-3.16 (m, 2H), 3.03 (t, *J* = 6.2 Hz, 1H), 1.36 (d, *J* = 15.8 Hz, 9H). ESI-HRMS calcd for $C_{23}H_{32}N_{13}O_6$ [M+H]$^+$: 586.2593, found: 586.2638.

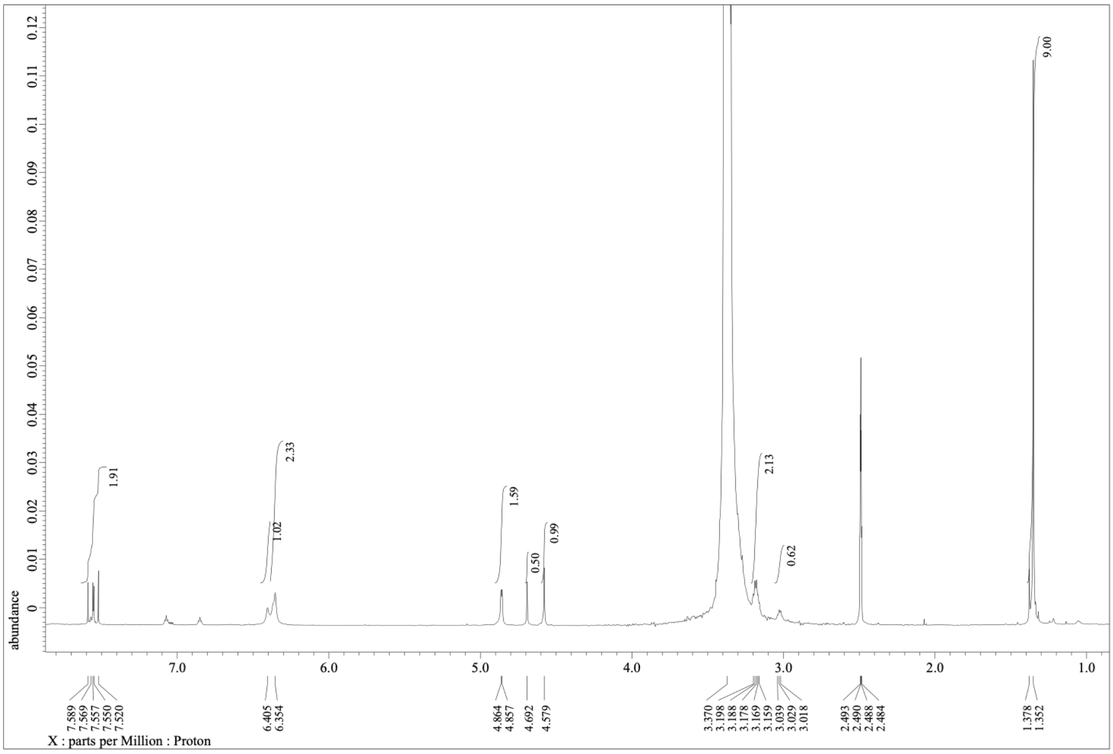

^{1}H NMR spectrum of **25**

2-(2-Amino-6-oxo-1*H*-purin-9(6*H*)-yl)-*N*-(2-(2-(2-amino-6-oxo-1*H*-purin-9(6*H*)-yl)acetamido)ethyl)-*N*-(2-aminoethyl)acetamide (26)

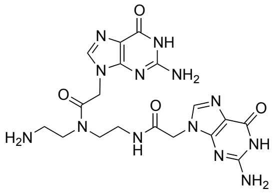

Compound **25** (15 mg, 0.026 mmol) was added HCl in 1,4-dioxane (4 M, 5 mL) and stirred at rt for 4 h. The reaction mixture was concentrated under reduced pressure, and the residue was dried by codistillation with MeOH. The residue was purified by HPLC (LC-2000Plus (JASCO); Discovery BIO Wide Pore C18 HPLC Column (Supelco), 21.2 × 250 mm, 10 µm; Flow rate: 10 mL/min; rt; Mobile phase A: 0.1% TFA in H_2O, B: 0.1% TFA in ACN; B 10% to 60 % (30 min); Detection : 260 nm) to afford **26** (4.4 mg, 9.1 µmol, 35% yield) as a pale yellow solid.

^{1}H NMR (600 MHz, DMSO-*d*$_6$) δ 10.70 (d, *J* = 6.2 Hz, 2H), 8.39 (t, *J* = 5.5 Hz, 1H), 7.68 (dd, *J* = 11.7, 4.1 Hz, 4H), 6.47 (d, *J* = 13.8 Hz, 1H), 6.41 (s, 3H), 4.90 (s, 1H), 4.71 (s, 1H), 4.60 (d, *J* = 20.0 Hz, 1H), 2.91 (q, *J* = 6.0 Hz, 1H), 2.45 (t, *J* = 1.7 Hz, 1H); ^{13}C NMR (151 MHz, DMSO-*d*$_6$) δ 167.7, 167.2, 166.8, 158.6, 158.4, 158.1, 157.9, 156.6, 153.9, 153.8, 151.5, 151.4, 138.7, 138.3, 119.8, 117.8, 115.8, 115.2, 115.1, 115.0, 115.0, 113.9, 48.7, 46.2, 46.0, 45.2, 45.0, 43.9, 43.6, 37.2, 37.0, 36.4, 35.4. ESI-MS : 486.2217 [M+H]$^+$.

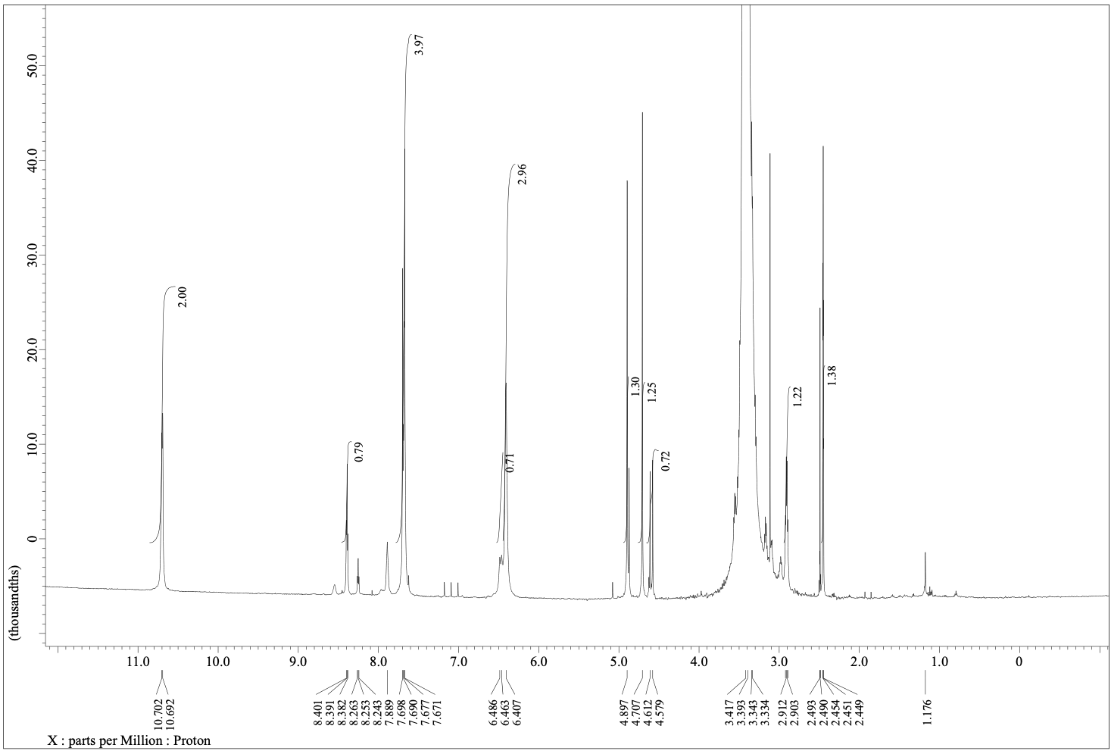

^{1}H NMR spectrum of **26**

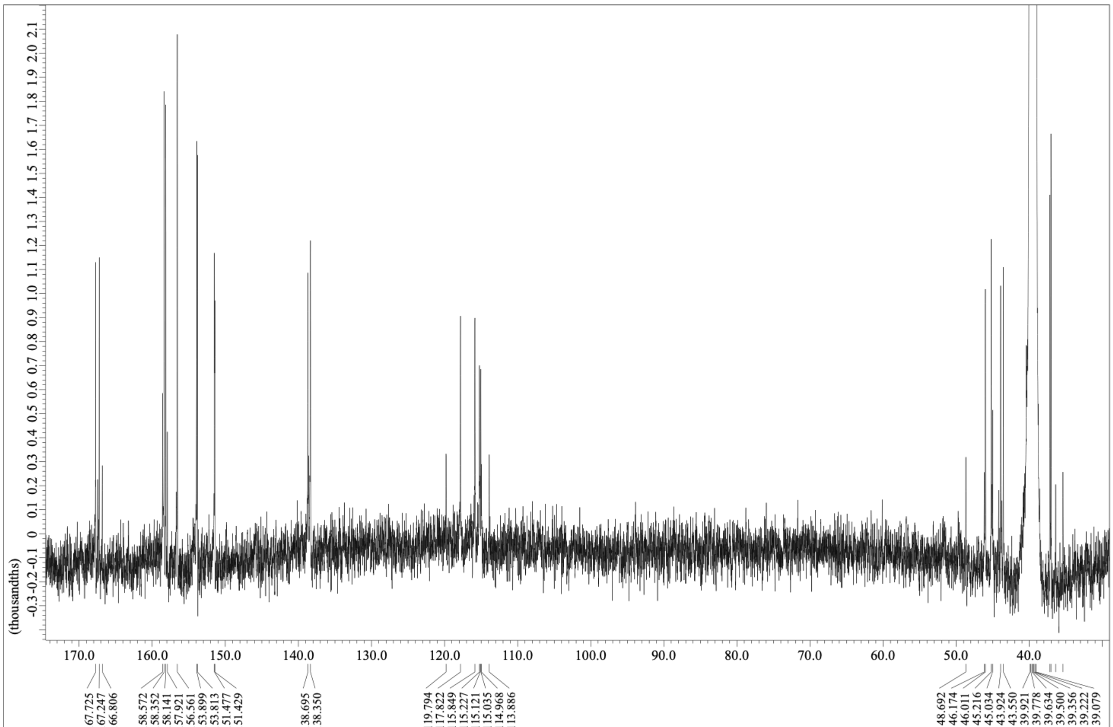

^{13}C NMR spectrum of **26**

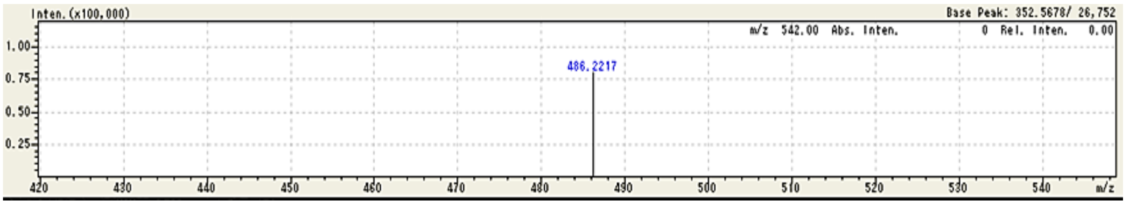

ESI-MS spectrum of **26**

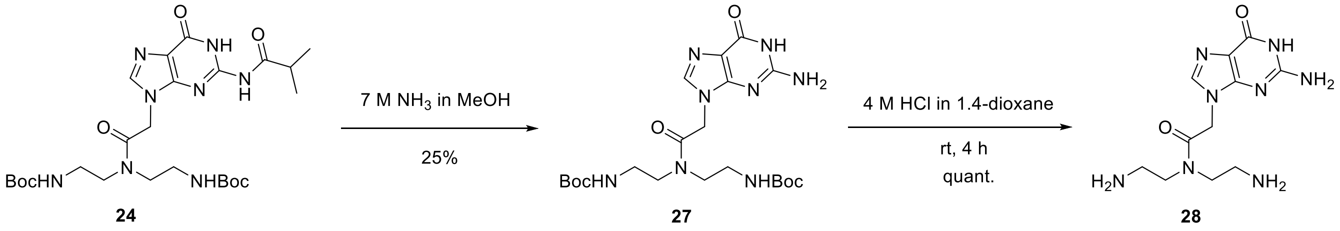

Scheme S9. Synthesis of compound **28**

Di-*tert*-butyl (((2-(2-amino-6-oxo-1*H*-purin-9(6*H*)-yl)acetyl)azanediyl)bis (ethane-2,1-diyl))dicarbamate (27)

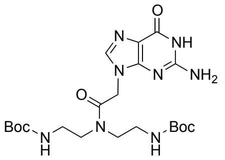

Compound **24** (72 mg, 0.13 mmol) was added NH_3 in MeOH (7 M, 5 mL), stirred for 38 h at rt. The reaction mixture was concentrated under reduced pressure, dried under vacuum. The residue was purified by flash silica gel column chromatography (Yamazen inject column size: S, Yamazen Hi-flash column size: M, CH_2Cl_2/MeOH = 100:0 to 50:50) to afford **27** (23.6 mg, 0.048 mmol, 37% yield) as an orange solid.

^{1}H NMR (600 MHz, CD_3OD) δ 7.67 (s, 1H), 5.01 (s, 2H), 3.56 (t, *J* = 6.2 Hz, 2H), 3.44 (t, *J* = 6.2 Hz, 2H), 3.36 (t, *J* = 6.5 Hz, 2H), 3.21 (t, *J* = 6.2 Hz, 2H), 1.47-1.41 (m, 18H). ESI-HRMS calcd for $C_{21}H_{35}N_8O_6$ $[M+H]^+$: 495.2674, found: 495.2683.

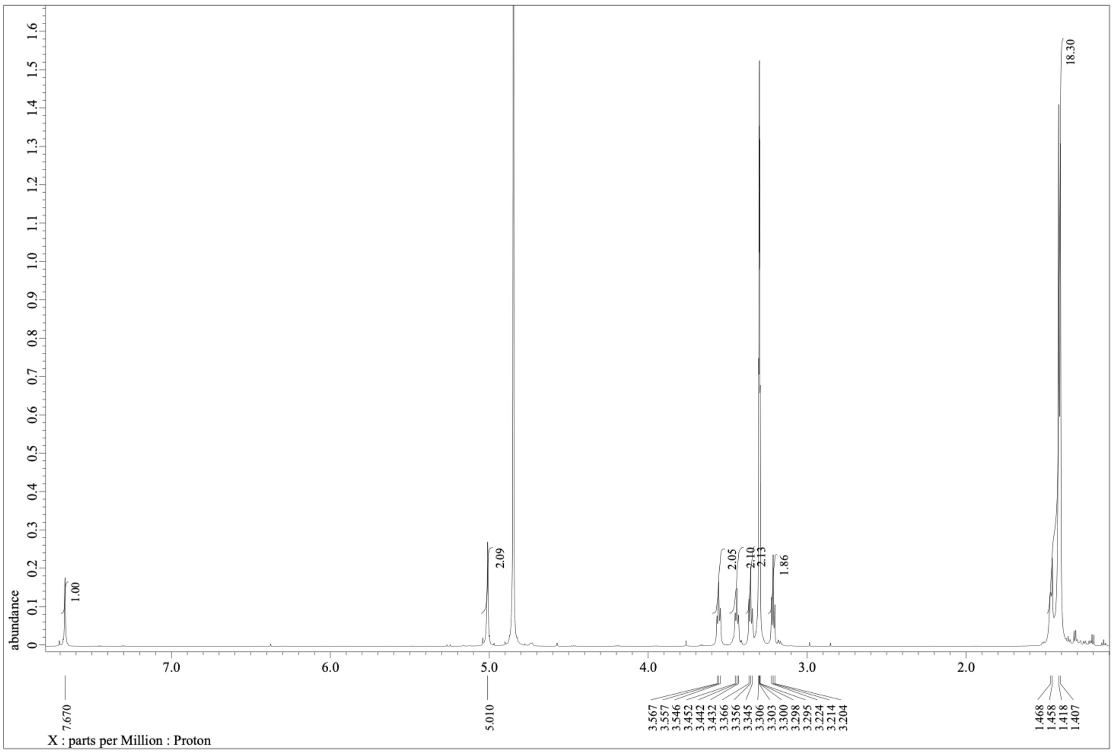

^{1}H NMR spectrum of **27**

2-(2-Amino-6-oxo-1*H*-purin-9(6*H*)-yl)-*N*,*N*-bis(2-aminoethyl)acetamide trihydrochloride (28)

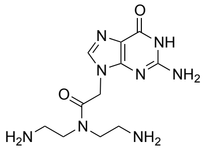

Compound **27** (24 mg, 0.049 mmol) was added HCl in 1,4-dioxane (4 M, 5 mL) and stirred for 4 h. The reaction mixture was concentrated under reduced pressure, and the residue was dried by codistillation with MeOH to afford **28** (25.3 mg, 0.069 mmol, quant.) as a pale orange solid.

^{1}H NMR (600 MHz, DMSO-d_6) δ 11.06 (s, 1H), 8.33 (s, 1H), 7.84 (s, 3H), 7.49 (s, 3H), 6.62 (s, 2H), 4.58 (s, 2H), 2.94 (t, *J* = 5.9 Hz, 2H), 2.81 (dt, *J* = 23.9, 5.3 Hz, 1H), 2.51 (d, *J* = 5.5 Hz, 2H), 2.31 (d, *J* = 5.5 Hz, 2H). ESI-MS: 294.9619 [M+H]$^+$.

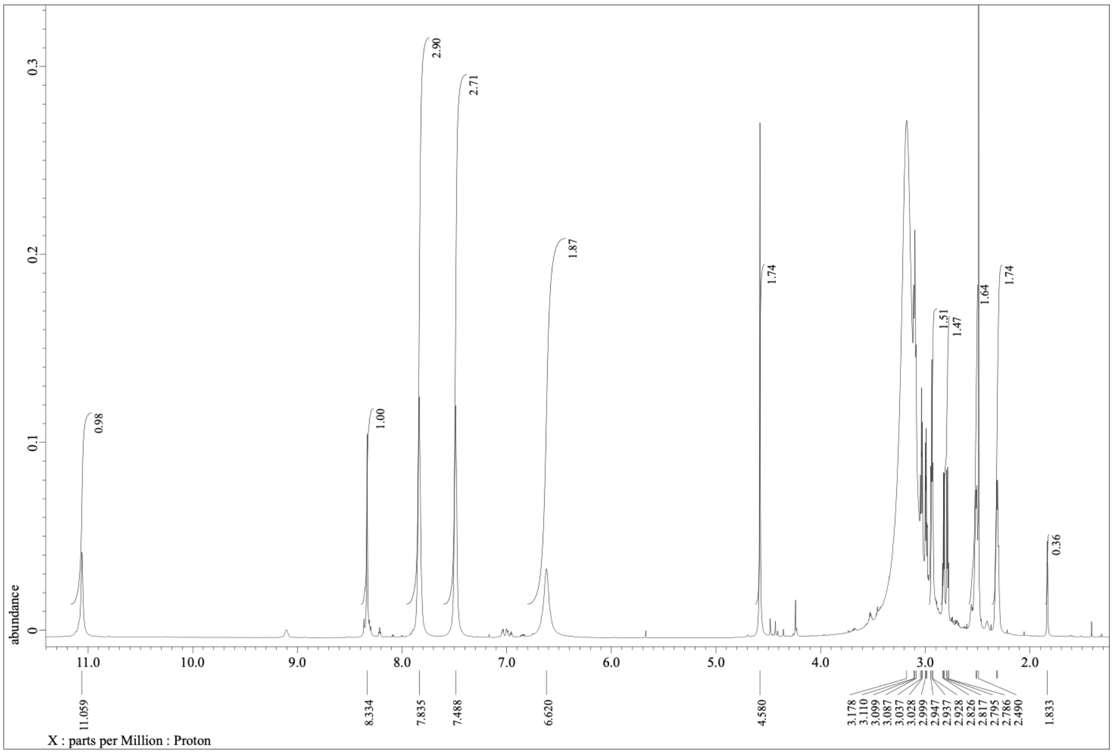

^{1}H NMR spectrum of **28**

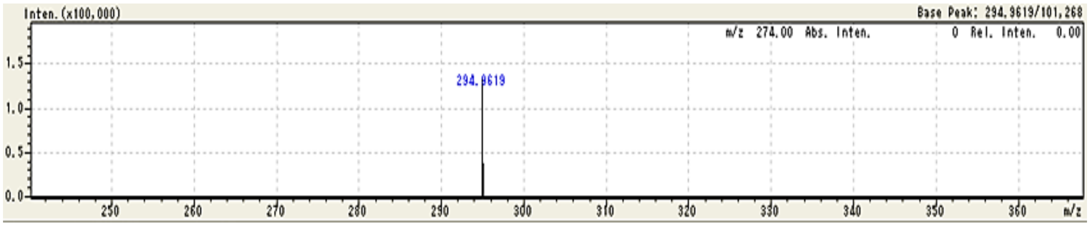

ESI-MS chromatogram of **28**

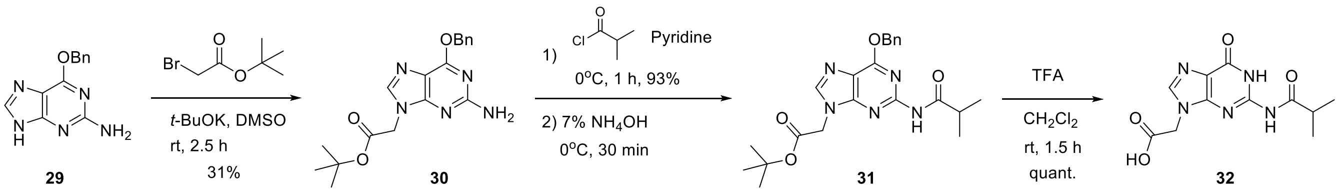

Scheme S10. Synthesis of compound **32**

***tert*-Butyl 2-(2-amino-6-(benzyloxy)-9*H*-purin-9-yl)acetate (30)**

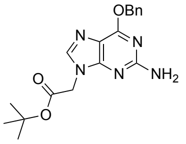

2-Amino-6-benzyloxypurine **29** (10 g, 41 mmol) was dissolved in DMSO (82 mL) and stirred at 0ºC. The mixture was added *t*-BuOK (5.58 g, 1.2 eq) slowly and stirred for 30 min at 0ºC. *tert*-Butyl bromoacetate (8.5 mL, 1.4 eq) was added dropwise and stirred for 1 h at rt. The reaction mixture was diluted with EtOAc, washed with sat. NH_4Cl aq., H_2O and brine. The organic layer was dried over Na_2SO_4, filtered, concentrated under reduced pressure. The residue was purified by flash silica gel column chromatography (Yamazen inject column size: 2L, Hi-flash column size: 3L, CH_2Cl_2/MeOH = 100:0 to 98:2) to afford **30** (4.6 g, 13 mmol, 31% yield) as a white solid.

^{1}H NMR (600 MHz, DMSO-*d*$_6$) δ 7.82 (s, 1H), 7.49 (d, *J* = 7.2 Hz, 2H), 7.39 (t, *J* = 7.2 Hz, 2H), 7.34 (t, *J* = 6.5 Hz, 1H), 6.50 (s, 2H), 5.49 (s, 2H), 4.79 (s, 2H), 1.40 (s, 9H). ESI-HRMS calcd for $C_{18}H_{22}N_5O_3$ [M+H]$^+$: 356.1717, found: 356.1733.

The position of *N*-alkylation was confirmed by 2D-NMR (HMBC) measurements (Correlation between C-4 and H-10).

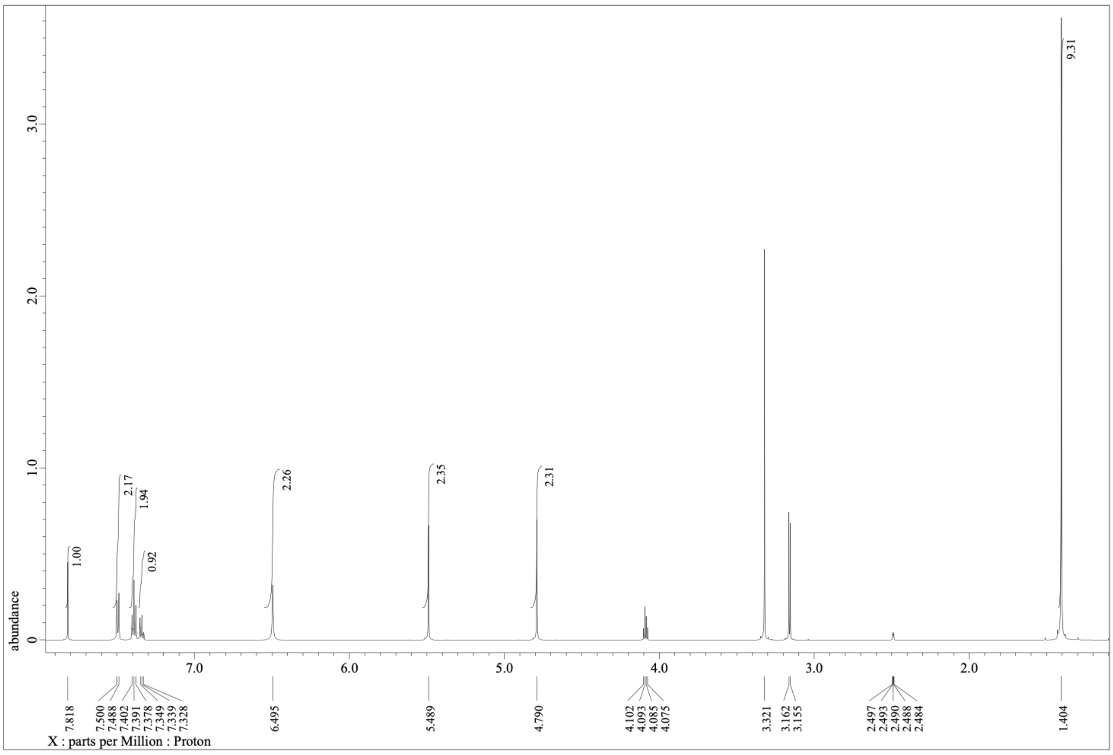

^{1}H NMR spectrum of **30**

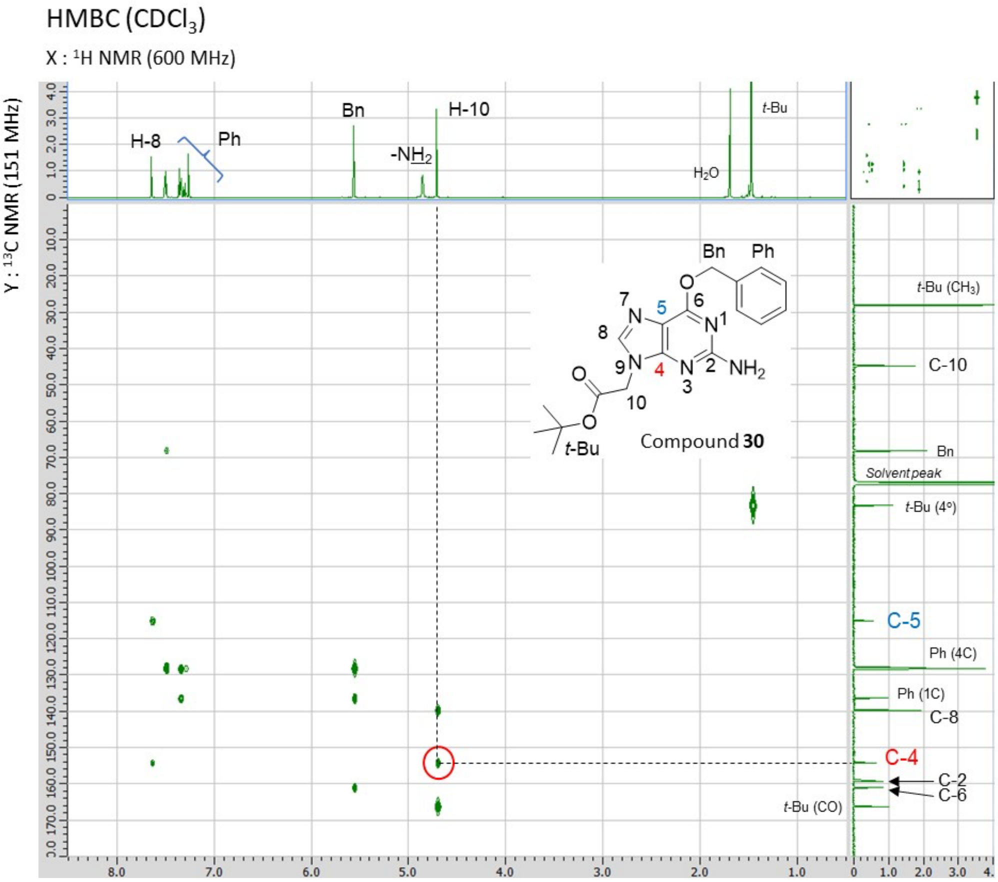

HMBC spectrum of **30**

tert-Butyl 2-(6-(benzyloxy)-2-isobutyramido-9_H_-purin-9-yl)acetate (31)

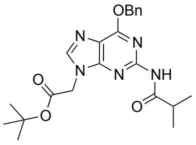

Compound **30** (4.6 g, 13 mmol) was dissolved in pyridine (65 mL) and stirred at 0ºC. Isobutyryl chloride (2.7 mL, 2 eq) was added dropwise then stirred for 2 h at rt. After stirring, the reaction mixture was added isobutyryl chloride (1.4 mL, 1 eq) at 0ºC, then stirred for 1 h at rt. After cooling to 0ºC, the reaction mixture was added dropwise ice-cold 7% NH_4OH (60 mL) over 3 min under well-stirring. The reaction mixture was kept stirring for 30 min at 0ºC. The reaction mixture was diluted with EtOAc, washed with sat. $NaHCO_3$ aq. and brine. The organic layer was dried over Na_2SO_4, filtered, concentrated under reduced pressure, dried under vacuum to afford **31** (5.1 g, 12 mmol, 93%) as a white foam solid.

^{1}H NMR (600 MHz, DMSO-d_6) δ 10.38 (s, 1H), 8.18 (s, 1H), 7.56 (d, J = 6.9 Hz, 2H), 7.40-7.33 (m, 3H), 5.62 (s, 2H), 4.94 (s, 2H), 3.16 (d, J = 4.8 Hz, 1H), 2.96-2.91 (quint., J = 6.8 Hz, 1H), 1.41 (s, 9H), 1.08 (d, J = 6.9 Hz, 6H), 0.97 (d, J = 6.9 Hz, 1H). ESI-HRMS calcd for $C_{22}H_{28}N_5O_4$ [M+H]$^+$: 426.2136, found: 426.2144.

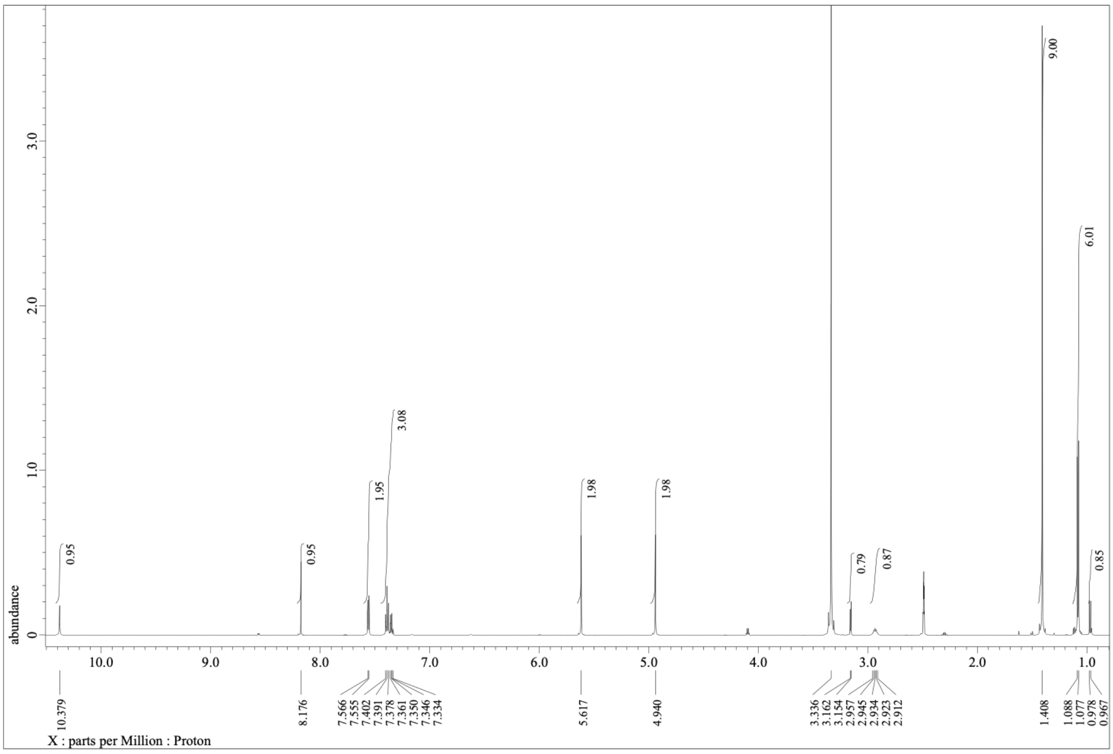

^{1}H NMR spectrum of **31**

2-(2-Isobutyramido-6-oxo-1*H*-purin-9(6*H*)-yl)acetic acid (32)

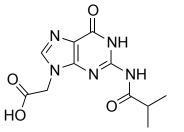

Compound **31** (5.1 g, 12 mmol) was dissolved in CH_2Cl_2 (10 mL) and TFA (40 mL) then stirred for 2 h at rt. The reaction mixture was diluted with toluene, concentrated under reduced pressure to remove the volatiles. The residue was added Et_2O, and triturated to solidify. The resulting precipitate was collected by filtration, washed with Et_2O, dried under vacuum to afford **32** (3.9 g, 14 mmol, quant.) as a white powder.

^{1}H NMR (600 MHz, DMSO-*d$_6$*) δ 12.08 (s, 1H), 11.66 (s, 1H), 7.95 (s, 1H), 4.89 (s, 2H), 2.78-2.73 (sept., *J* = 6.9 Hz, 1H), 1.10 (d, *J* = 6.9 Hz, 6H). ESI-HRMS calcd for $C_{11}H_{14}N_5O_4$ [M+H]$^+$: 280.1040, found: 280.1040.

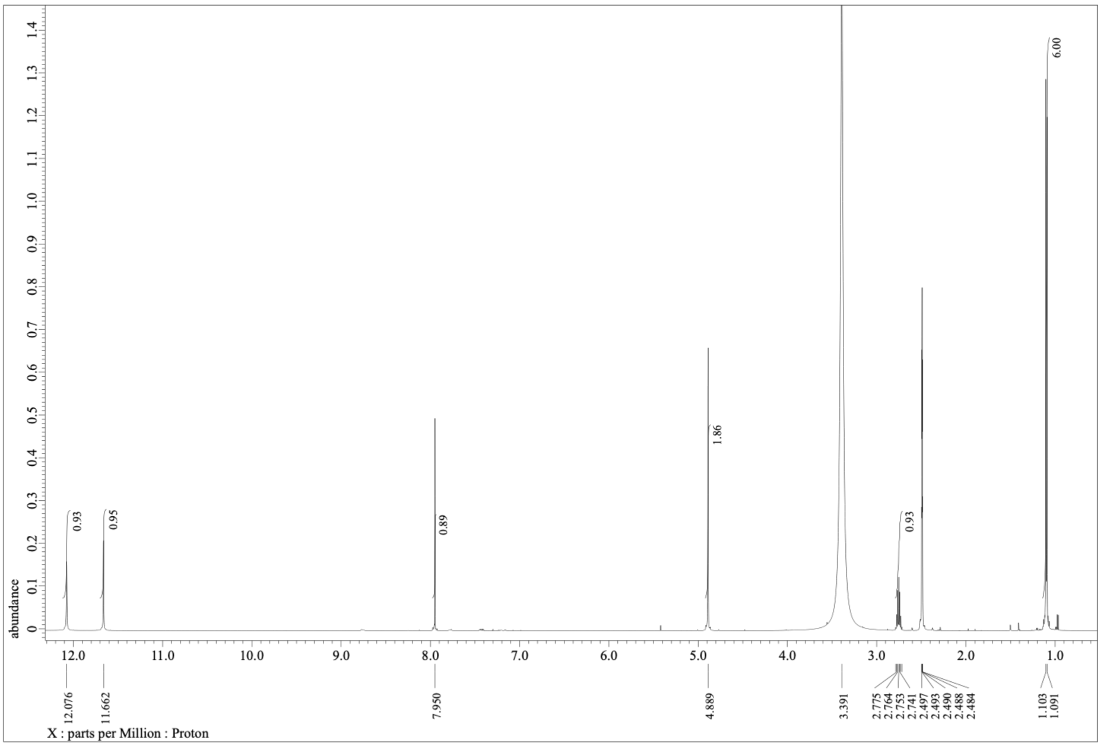

^{1}H NMR spectrum of **32**

References

[S1] K. Ikeda, Y. Yanase, K. Hayashi, Y. Hara-Kudo, G. Tsuji, and Y. Demizu, *Bioorg. Med. Chem. Lett.* **2021**, 32, 127713, DOI: 10.1016/j.bmcl.2020.127713.

[S2] Y. Yang, X. Wu, N. Busschaert, H. Furuta, P. A Gale, *Chem. Commun.* **2017**, 53, 9230-9233, DOI: 10.1039/c7cc04912a.

[S3] D. D. N'Da, E. Neuse, M. Nell, C. E. J. van Rensburg, *S. Afr. J. Chem.* **2003**, 59, 33-42.
